# Synthetic antibacterial minerals: harnessing a natural geochemical reaction to combat antibiotic resistance

**DOI:** 10.1038/s41598-022-05303-x

**Published:** 2022-01-24

**Authors:** Keith D. Morrison, Kelly A. Martin, Josh B. Wimpenny, Gabriela G. Loots

**Affiliations:** 1 Nuclear and Chemical Sciences Division, Physical and Life Sciences, Lawrence Livermore National Laboratory, Livermore, CA 94550 USA; 2 Biosciences and Biotechnology Division, Physical and Life Sciences, Lawrence Livermore National Laboratory, Livermore, CA 94550 USA

**Keywords:** Pathogens, Drug development

## Abstract

The overuse of antibiotics in clinical and livestock settings is accelerating the selection of multidrug resistant bacterial pathogens. Antibiotic resistant bacteria result in increased mortality and financial strain on the health care and livestock industry. The development of new antibiotics has stalled, and novel strategies are needed as we enter the age of antibiotic resistance. Certain naturally occurring clays have been shown to have antimicrobial properties and kill antibiotic resistant bacteria. Harnessing the activity of compounds within these clays that harbor antibiotic properties offers new therapeutic opportunities for fighting the potentially devastating effects of the post antibiotic era. However, natural samples are highly heterogenous and exhibit variable antibacterial effectiveness, therefore synthesizing minerals of high purity with reproducible antibacterial activity is needed. Here we describe for the first time synthetic smectite clay minerals and Fe-sulfide microspheres that reproduce the geochemical antibacterial properties observed in natural occurring clays. We show that these mineral formulations are effective at killing the ESKAPE pathogens (*Enterococcus* sp.,* Staphylococcus aureus, Klebsiella pneumoniae, Acinetobacter sp., Pseudomonas aeruginosa and Enterobacter* sp.) by maintaining Fe^2+^ solubility and reactive oxygen species (ROS) production while buffering solution pH, unlike the application of metals alone. Our results represent the first step in utilizing a geochemical process to treat antibiotic resistant topical or gastrointestinal infections in the age of antibiotic resistance.

## Introduction

A resurgence of inquiry into alternative antibacterial mechanisms has emerged as human pathogens are continuing to evolve antibiotic resistance. Documented use of reduced metal-rich clays in healing necrotizing fasciitis has led to a renewed interest in ancient uses of minerals for healing wounds^1^. Research on the first antibacterial clay deposit in the U.S. (near Crater Lake, Oregon) provided a basic understanding of the role minerals can play in killing antibiotic resistant pathogens^1–4^. The most antibacterial mineral assemblages observed in nature provided the extended release of Fe^2+^ and reactive oxygen species (ROS) while buffering solution pH to 5–4^2–5^. The sustained release of Fe^2+^ from natural antibacterial minerals was found to cause the oxidation of bacterial membranes while generating excess intracellular Fe that oxidized bacterial DNA while precipitating iron-oxide nanoparticles^5^. Two minerals (smectite and pyrite) were found to control the antibacterial activity of natural clays. The smectite clay minerals sustained hydration of clay poultices while providing cation exchange capacity, pH buffering, and stabilization of Fe^2+^ in interlayer sites^2,3,5^. The Fe-sulfide minerals showed increased reactivity and ROS generation as particle size decreased^3,4^. This geochemical mechanism may be especially useful for treating topical wounds infected with antibiotic resistant bacteria, that shift to alkaline pH and result in localized tissue hypoxia^6–8^. Additionally, these synthetic minerals may also aid in treating gastrointestinal infections where bacterial toxins result in dysentery^9^, by killing the invading pathogen while adsorbing and breaking down toxins.

While the scientific pursuit of natural antibacterial clays has emerged in the last 15 years, along with an exponential increase in publications on natural antibacterial clays (Fig. S1), the promise of these clays as a new generation of antimicrobials has not yet come to fruition due to the highly heterogenic nature of natural clays without consistent reliable bioactive properties. Toward this end, synthetic versions of natural antibacterial minerals with high chemical purity and reproducible antibacterial activity are required for use in clinical settings. Our research introduces for the first-time methodologies for generating synthetic minerals that produce controlled geochemical reactions that can be harnessed to eradicate antibiotic resistant bacteria. We show that these synthetic minerals can be highly potent in killing ESKAPE pathogens within 24 h of administration, therefore representing novel mineral-based therapeutics for the eradication of bacteria that currently evade available antibiotic treatments.

## Results and discussion

**Figure 1:**
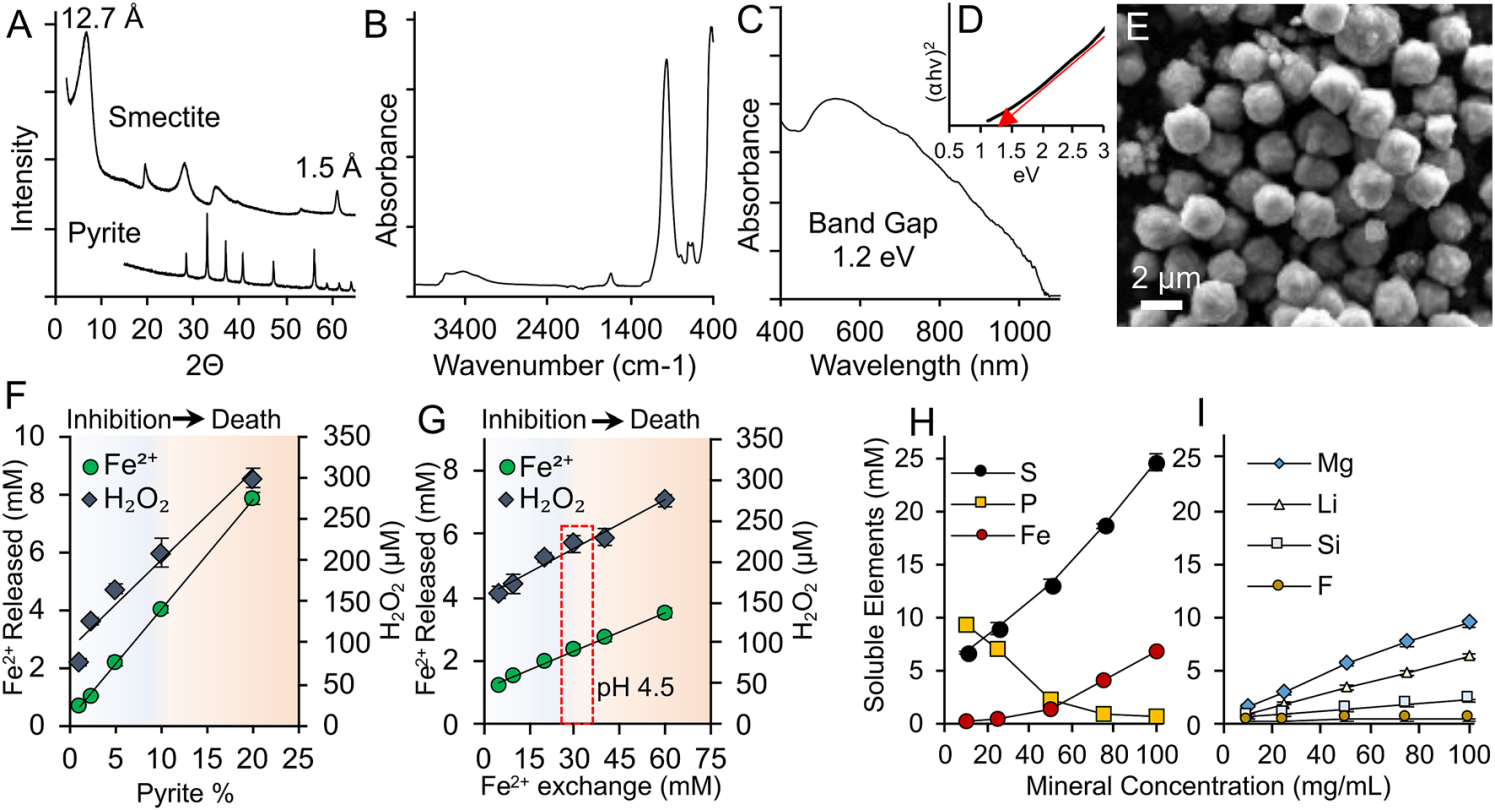
Synthesis and tuning of antibacterial mineral activity. **(A)** XRD patterns of synthetic smectite and pyrite showing single mineral phases. **(B)** FTIR spectrum of synthetic smectite. **(C,D)** Band-gap measurements of synthetic pyrite. **(E)** SEM images of synthetic pyrite particles, showing micro-spheres with a 1–2 µm size range. **(F,G)** Antibacterial activity of antibacterial mineral mixtures (50 mg/mL) tested against *E. coli* in TSB media for 4 h while **(F)** varying pyrite concentration (1–20 wt.%) with a fixed concentration of Fe^2+^ exchanged solutions (30 mM FeSO_4_) or **(G)** a fixed pyrite concentration (5 wt.%) and a varied concentration of Fe^2+^ exchange solutions (5–60 mM FeSO_4_). Variations in the concentration of pyrite or Fe^2+^ exchange solutions can be used to control the release of Fe^2+^ and H_2_O_2_ and antibacterial activity of the mineral composites. (**G**, red box) A mineral mixture containing 95% smectite, 5% pyrite and exchanged with 30 mM Fe^2+^ mimicked the antibacterial activity of natural antibacterial minerals that maintained Fe^2+^ and H_2_O_2_ generation. **(H,I)** ICP-MS elemental analysis of soluble elements leached form the antibacterial minerals in TSB media after 24 h. **(H)** The release of Fe and S increase in a dose dependent manner, while P from the growth media decreased with increasing mineral concentrations. **(I)** Concentrations of Mg, Li, Si and F also increase with mineral concentration and relate to a stoichiometric dissolution of the smectite during the reaction. Minimum bactericidal concentrations (MBC) concentrations of Fe^2+^, Mg, Li, Si and F were also measured (Table S2).

The rapid synthesis of smectites with a hectorite composition was achieved in 5 days utilizing hydrothermal pressure bombs with fumed silica nanoparticles, MgOH_2_ and LiF. The X-ray diffraction (XRD) and Fourier transform infrared spectroscopy (FTIR) measurements revealed that only smectite minerals formed (Fig. 1A,B, S2 and S3; Table S1). Elemental analysis (X-ray fluorescence) of the smectites revealed < 0.016% chemical impurities arising from Cu and Ni. Additionally, the mineral formula is consistent with the expected structure of hectorite clay minerals. The addition of fluorine to the reaction greatly accelerated the smectite crystallization rate and attempts to synthesize smectites (in 5 days) without fluorine failed (Fig. S2). The resulting smectites are 99.9% chemically pure and eliminate toxic metals that may be present in natural samples. The synthetic smectites have a particle size of 355 ± 32 nm and a cation exchange capacity of 98.9 ± 3.1 meq/100 g (Fig. S4). Smectite clays maintain a net negative charge on the basal surfaces across a wide pH range^10^. Zetapotential measurements show that the synthetic smectites maintain a negative zetapotential of -22 to -30 from pH 3 to 9, respectively (Fig. S4).

Synthetic smectite chemical formula: Na_0.33_(Mg_2.69_, Li_0.61_)_3_(Si_4.04_O_10_)(F_0.75_,OH_1.25_)_2_.

Iron (Fe)-sulfide micro-spheres (pyrite) were also synthesized with high chemical and mineralogical purity using polysulfides, polyvinylpyrolidone and Fe^2+^ (Fig. 1A and S5). Solvent washes of the pyrite micro-spheres removed unreacted S^0^ impurities, leaving only pyrite as the final product (Figs. S5 and S6). The purified pyrite contained 53.9 and 46.1 atom % S and Fe (respectively), which agrees with the predicted ratio for pure pyrite. Only minor (< 0.01 wt.%) chloride impurities were found in the synthetic pyrites using XRF. The band gap of the synthetic pyrite was 1.2 eV (Fig. 1C,D), which gives the mineral semi-conductor properties that are linked to reactive oxygen species (ROS) generation in solution^3,11^. The particle size of the synthetic pyrite was 1–2 microns in diameter which mimics the size range of the most antibacterial natural samples (Fig. 1E and S6)^3,4^.

Initial mixtures of the synthetic smectite and pyrite micro-spheres with 5–10% pyrite (range observed in natural samples) showed no antibacterial activity, Fe^2+^ release or H_2_O_2_ generation when tested against an *E. coli* strain (ATCC 25922) (Figs. S7 and S8). Previous studies hypothesized that the antibacterial activity of the natural samples resulted from smectite cation exchange of Fe^2+^ coupled to redox cycling and pyrite oxidation when samples were re-hydrated^2–4^. We found that exchanging the samples with Fe^2+^ provided the rapid release of a soluble pool of Fe^2+^ that establishes redox cycling with pyrite^2,3,11^. The smectite interlayer surfaces have a net negative charge (Fig. S4) that participates in cation exchange reactions that can be utilized to regulate the antibacterial activity of the mineral mixtures. By altering the concentration of pyrite (Fig. 1G) or the amount of Fe^2+^ exchanged with the smectites (Fig. 1H), we were able to control the solution pH, release of Fe^2+^ and generation of ROS to reach levels that were bactericidal (see SI for extended discussion). The tunable properties of the antibacterial minerals can be used to generate formulations that inhibit bacterial growth or become bactericidal (Fig. 1F,G).

Antibacterial mineral formulations containing 95 wt.% smectite and 5 wt.% pyrite, exchanged with 30 mM Fe^2+^SO_4_ mimicked the metal release, hydrogen peroxide generation and pH observed in natural antibacterial samples^2,3,5^, while titrating the system with the lowest concentration of elements for bactericidal activity to occur. This extended release of Fe^2+^and ROS, along with pH buffering, is not possible when metal solutions alone are used, due to the rapid oxidation rate of Fe^2+^ at pH 4–6 (~ 3 × 10^–5^ mol·L^−1^·min^−1^)^5,12^. The release of Fe, S, Mg, Li, Si and F form the minerals increased linearly with increasing mineral concentration, which allowed a predictable dose response to be established (Fig. 1H,I). The dissolution of the smectite minerals was observed but accounted for less than 1 wt.% of the added smectite. Minimum bactericidal concentrations (MBC) of the elements leached from the smectite were measured and were not released at sufficient concentrations to cause bacterial cell death alone (Table S2). This suggests that the sustained release of Fe^2+^, ROS and pH buffering were responsible for the antibacterial activity and was analogous to the antibacterial mechanism observed in natural samples^2–5^.

**Figure 2:**
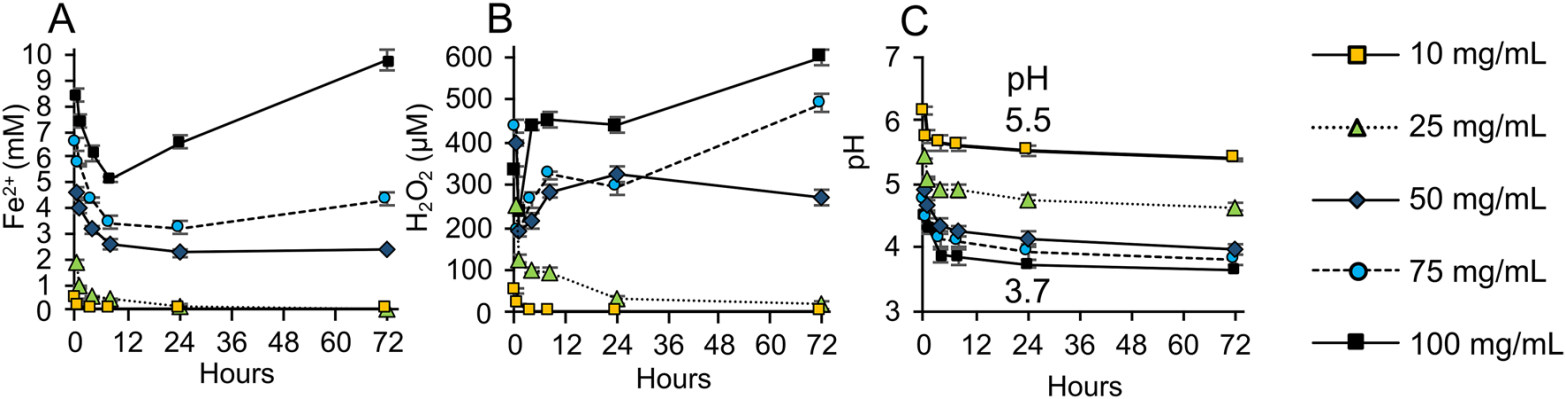
Ferrous iron release, ROS generation and pH buffering. All experiments were performed with a mineral mixture containing 95 wt. % smectite and 5% pyrite, exchanged with 30 mM FeSO_4_. **(A)** Concentrations of Fe^2+^ released from different doses of minerals (10–100 mg/mL) in TSB media over 72 h. **(B)** Concentrations of H_2_O_2_ released from minerals in TSB media over 72 h. **(C)** Changes in pH over 72 h.

**Figure 3:**
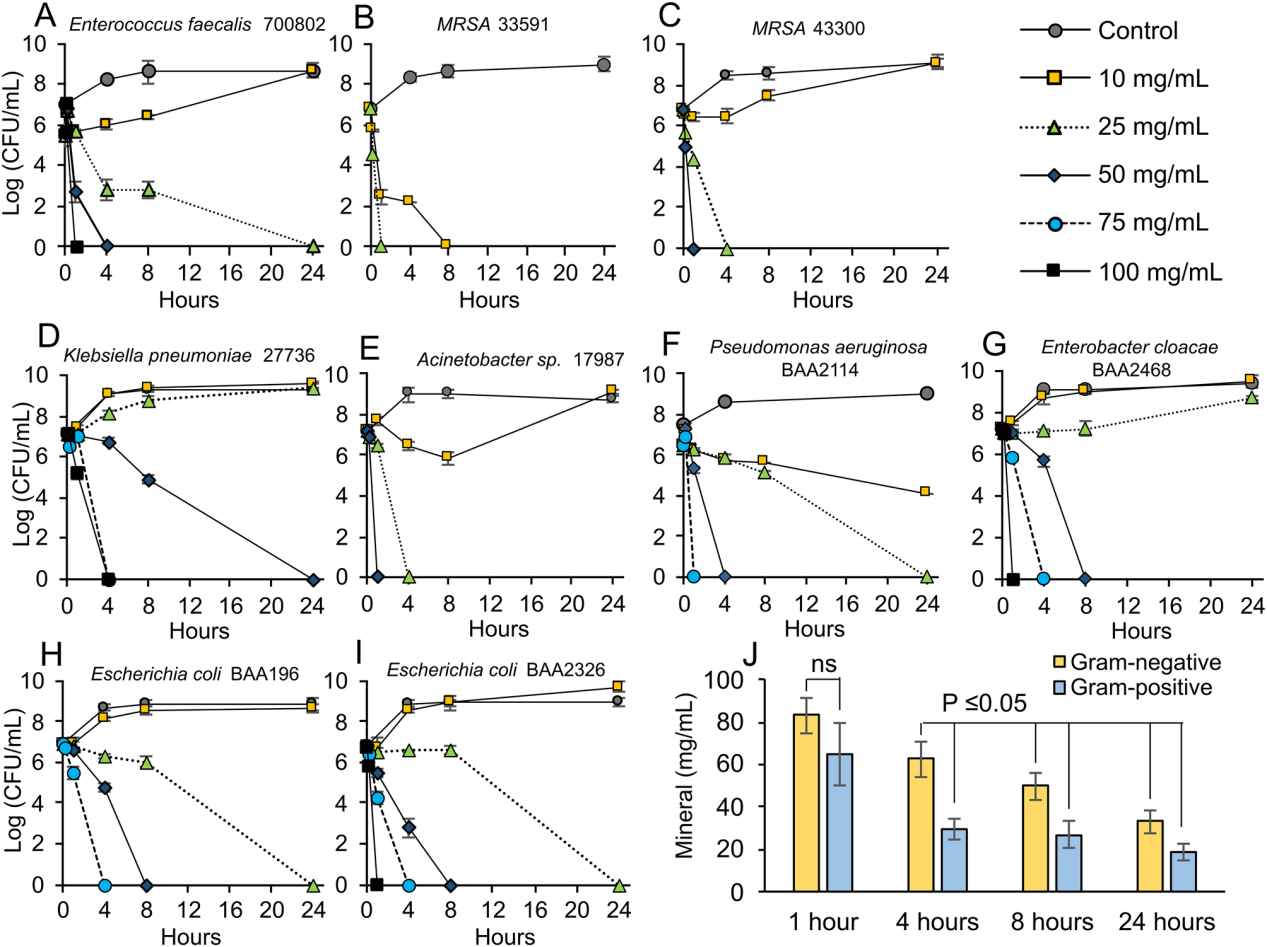
Antibacterial susceptibility testing of the multi-drug resistant ESKAPE pathogens. All experiments were performed with a mineral mixture containing 95 wt.% smectite and 5% pyrite, exchanged with 30 mM FeSO_4_. **(A–I)** Antibacterial susceptibility testing of antibiotic resistant ESKAPE pathogens to determine the dose response (10–100 mg/mL) where antibacterial activity occurs over 24 h. **(J)** Average MBC dose for Gram-negative or positive pathogens measured over 24 h.

Understanding how these geochemical processes influenced the reactivity of the synthetic antibacterial minerals allowed us to establish a dose response for the ESKAPE pathogens (*Enterococcus *sp., *Staphylococcus aureus*, *Klebsiella pneumoniae*, *Acinetobacter sp.*, *Pseudomonas aeruginosa* and *Enterobacter *sp.), that represent the most deadly antibiotic resistant hospital acquired infections^13^. We tested 11 strains of antibiotic resistant and sensitive bacteria (Table S3) with synthetic antibacterial mineral mixtures (containing 95 wt.% smectite with 5 wt.% pyrite and exchanged with 30 mM Fe^2+^SO_4_; Fig. 1G red square) at mineral concentrations ranging from 10 to 100 mg/mL (Fig. 2, Table S3). The release of Fe^2+^ and H_2_O_2_ (in TSB media) was maintained for > 24 h when mineral concentrations were > 25 mg/mL and the pH was buffered between 5.5 and 3.7 (Fig. 2). The results indicate that Gram-positive bacteria were eradicated at a lower dose (after 4 h) when compared to Gram-negative (Fig. 3J). This may be due to differences in bacterial Fe^2+^ uptake and efflux mechanisms with varying expression levels^14,15^. A 50 mg/mL dose was capable of killing all bacterial pathogens after 24 h with many strains dying within the first 4 h of the exposure (Fig. 3A–I). This dose coincides with the extended release of mM and µM levels of Fe^2+^ and H_2_O_2_, respectively (Fig. 2A,B). Ferrous sulfate solutions at similar concentrations were also tested for antibacterial activity and required higher concentrations to achieve bactericidal activity (Table S2) and rapidly oxidize in solution^5^.

**Figure 4:**
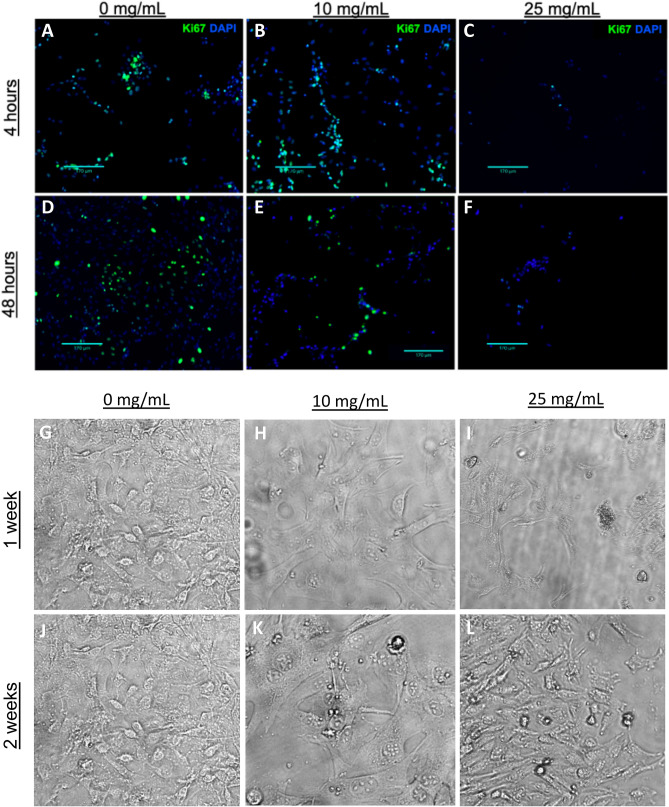
Mineral toxicity to fibroblasts. Ki67 staining denoting fibroblast proliferation following 4 h exposure to 0, 10 and 25 mg/mL of mineral mixtures (containing 95 wt.% smectite with 5 wt.% pyrite and exchanged with 30 mM Fe^2+^SO_4_) at 4 h post dose **(A–C)** and 48 h post dose **(D–F)**. Scale bars = 170 µm. Brightfield imaging (×10 magnification) of long-term recovery of fibroblasts following 4 h exposure to 0, 10 and 25 mg/mL of minerals at 1 week post exposure **(G–I)** and 2 weeks post mineral exposure **(J–L)**.

Mouse fibroblasts were exposed to 10 and 25 mg/mL mineral mixtures in RPMI media to determine if mammalian cells in culture could tolerate the Fe^2+^ and ROS release in addition to the subsequent drop in pH (Fig. 4 and S10). The fibroblast cells did experience low levels of cytotoxicity and some cell death was observed during the 4 h exposure to the synthetic antibacterial minerals, however upon switching to mineral-free media, cells recovered during the next 7 to 14 days. Toxicity assays of fibroblasts using the trypan blue assay indicated that ~ 62% of the cells remain viable after a 24 h exposure to a 100 mg/mL mineral suspension (Fig. S10). We postulate that synthetic clays may be able to promote healing by both killing the pathogenic bacterial and neutralizing the alkaline environment of chronic non-healing wounds.

Chronic non-healing wounds infected with antibiotic resistant bacteria produce a physiochemical environment that presents challenges for immune response and medical treatments. The surface layers of healthy skin are acidic, with pH values ranging from 4.0 to 6.3, and provide protection against bacterial infections and growth^6,16–20^. Chronic non-healing wounds shift to alkaline pH (8 to 9) and prevent the release of oxygen from hemoglobin in infected tissues, resulting in localized hypoxia that favors infection^6,18,21–23^. Additionally, the elevated pH and reducing environment (redox potential, Eh < −140 mV) in the wound environment make the respiratory burst of ROS used by macrophages and neutrophils to kill invading pathogens difficult to achieve^7,24^. There is growing evidence that the acidification of chronic wounds results in improved recovery outcomes^6,16,18,21,22,25,26^. However, the acidification of chronic wounds alone was not shown to effectively eliminate bacterial pathogens and in many cases the buffering capacity of the wound environment exceeded that of the acid solutions applied^6,21,26^. The application of synthetic antibacterial minerals may act in a manner that is analogous to the immune response, by engulfing bacterial pathogens in a mineral matrix that releases ROS while lowering pH and killing antibiotic resistant bacteria.

**Figure 5:**
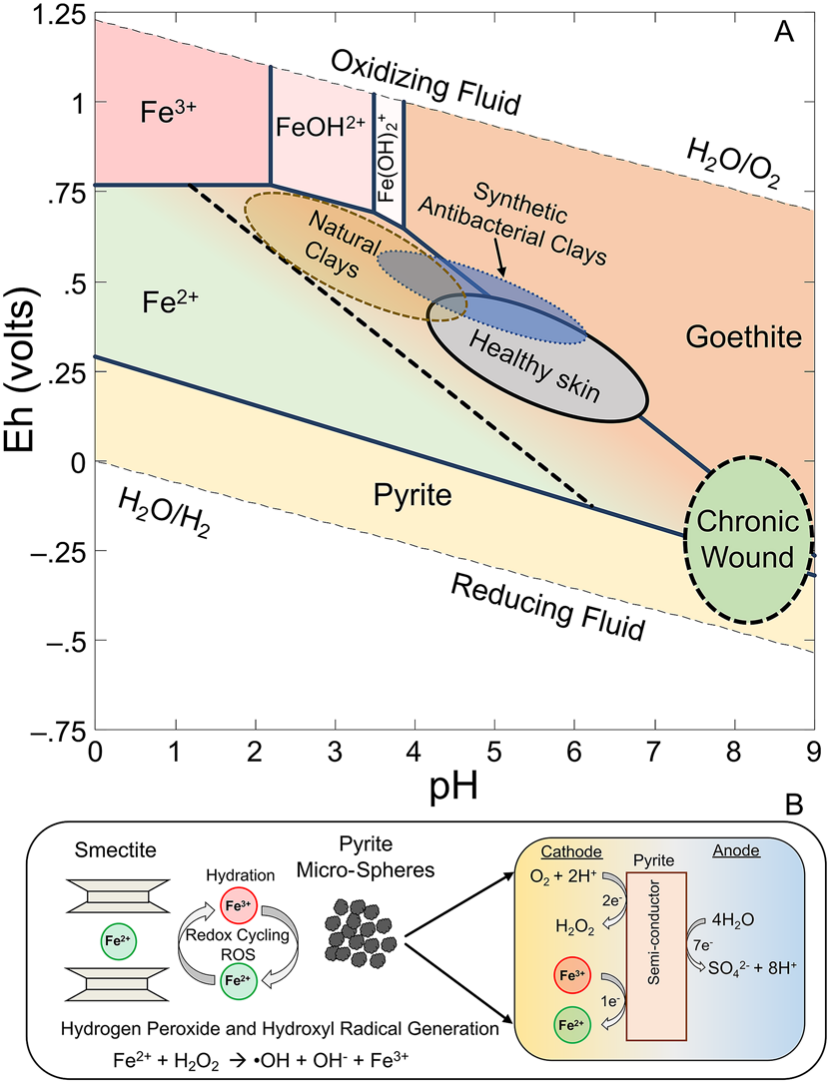
Pourbaix diagram of the Fe–S–O–H system and geochemical reactions that produce the antibacterial activity. **(A)** Plot of mineral and aqueous cation stability fields for Fe along with the pH and Eh ranges found in natural antibacterial minerals, synthetic antibacterial minerals, chronic wounds and healthy skin (see main text for references). The dashed line in the Fe^2+^ stability field represents the shift in goethite stability that occurs as Fe concentrations increase due to the oxidation of pyrite and cation exchange of smectite interlayer spaces. Once the mineral system reaches thermodynamic equilibrium in an oxidizing environment, Fe^3+^-oxides (goethite) will form along with the loss of antibacterial activity. **(B)** Antibacterial mechanism of synthetic antibacterial mineral systems, showing the interplay of smectite cation exchange coupled to pyrite oxidation and redox cycling to maintain the generation of soluble Fe^2+^ and reactive oxygen species.

Geochemical modeling of the pH and Eh stability fields of minerals in the Fe–S–O–H system can be used to provide context to the antibacterial reactions that are occurring, in relation to the treatment of chronic non-healing wounds (Fig. 5A). Pyrite is stable in reducing environments, however when the antibacterial minerals are hydrated the fluids shift towards a new oxidized equilibrium with the atmosphere. As pyrite oxidation and smectite interlayer Fe^2+^ release occurs, the pH and Eh of the fluids reacting with the antibacterial minerals shift into the goethite stability field. It is this transition from reducing to oxidizing fluids that imbues the minerals with antibacterial activity via the generation of ROS. Natural antibacterial minerals ranged in pH from pH 2–4.5 with Eh values ranging from + 400 to + 750 mV^2,3,5^. The Eh values of healthy oxygenated tissues are ≥  + 300 mV^23^. The majority of natural antibacterial minerals had pH values < 3 and released > 10 mM concentrations of Fe and Al^3^. While these pH, Eh and metal concentrations are effective at killing antibiotic resistant bacteria, they would result in greater toxicity to mammalian cells when applied topically. The synthetic mineral formulations can be tuned to have pH and Eh values ranging from 3.7 to 6.1 and + 300 to + 600, respectively and overlap with the pH and Eh ranges observed in human skin (Fig. 5A).

Synthetic antibacterial minerals represent a promising avenue to treat chronic non-healing wounds infected with antibiotic resistant bacteria in clinical settings. They work by maintain Fe^2+^ solubility from smectite interlayer exchange coupled to Fe^3+^ oxidation of the pyrite surface, subsequently forming Fe^2+^ (Fig. 5B). The pyrite acts as a semi-conductor and allows for the generation of H_2_O_2_ as O_2_ is reduced at mineral cathode sites^11^. This H_2_O_2_ then reacts with soluble Fe^2+^ and forms hydroxyl radicals. Once thermodynamic equilibrium is achieved, Fe^3+^-oxide minerals represent the stable mineral endmembers. This shift to equilibrium in an oxidizing environment was associated with a loss of antibacterial activity^3^, indicating that the transition from a reduced chemical equilibrium to an oxidizing environment is what drives the antibacterial activity of the minerals. This geochemical cycle has been shown to effectively kill antibiotic resistant bacteria and the production of mineralogically pure end members for this process will allow for clinical applications of antibacterial minerals.

## Materials and methods

### Synthesis of smectite clay minerals

Fluorohectorite smectite clay minerals were synthesized using methods modified from^27,28^. Brucite (Mg(OH)_2_) was precipitated by mixing 50 mL of 1 M NaOH with a 200 mL solution of MgCl_2_ (25 mg/mL). The solution was stirred for 5 min, transferred into a centrifuge tube and spun at 3500 rpm for 10 min. The supernatant was decanted and 3 g of fumed SiO_2_ (100–200 nm particle size) was added, followed by 0.4 g LiF. The suspension was then transferred from the centrifuge tube to a beaker and diluted to 300 mL, achieving a 3% solid to solution ratio. The suspension was stirred at 300 rpm for 30 min. The homogenized solution was transferred into a 400 mL polytetrafluoroethylene (PTFE) lined hydrothermal reactor and heated at 200 °C for 5 days. After 5 days the clays were cooled to room temperature and 150 mL was transferred to centrifuge tubes (250 mL volume) followed by the addition of 2 M NaCl to flocculate and exchange all the smectite surfaces with Na^+^ cations. Clays were centrifuged at 3500 rpm for 10 min, resuspended with 2 M NaCl (200 mL) and allowed to equilibrate for 30 min. The clays were pelleted by centrifugation and then transferred to dialysis tubing with a 5–8 K Dalton molecular weight cutoff. Excess salts were removed by placing the dialysis tubes in a constant flow DIW bath for 3 days. The removal of NaCl was verified by the AgNO_3_ precipitation method. The dialyzed clays were transferred to a PTFE beaker and dried at 65 °C, rehydrated with 50 mL DIW, freeze-dried and ground into a powder.

### Pyrite synthesis

Pyrite synthesis using only elemental sulfur (S^0^) as the sulfur source was carried out by mixing 5 g of S^0^ with 5 g FeCl_2_ and 5 g polyvinylpyrrolidone (PVP) in 110 mL of DIW. The suspension was then transferred into a PTFE lined hydrothermal reactor and heated at 200 °C for 48 h. After 48 h the samples were centrifuged and rinsed in DIW and 1 M HCl (2x) to remove Fe-oxide impurities^29^. However, this method left wt. % quantities of S^0^ in the final product and produced pyrite particles with variable size and morphology. To mitigate these problems, we developed a method to generate Fe-sulfide microspheres with similar size and morphology to natural antibacterial clays. The pyrite microspheres were synthesized using polysulfides and PVP. A polysulfide solution was prepared by dissolving 5 g of Na_2_S in 10 mL DIW, followed by the addition of 5 g elemental S^0^ and heating at 75 °C until a bright orange solution formed. A separate beaker with 5 g FeCl_2_ and 5 g polyvinylpyrrolidone (PVP) dissolved in 100 mL DIW was stirred for 5 min at 300 rpm and heated to 75 °C. The Fe^2+^-PVP solution was slowly added to the polysulfide solution and stirred at 300 rpm (75 °C) for 1 min. The solution was transferred into a 200 mL PTFE lined hydrothermal reactor and heated to 200 °C for 48 h. After the reaction was complete the reactor was cooled to room temperature and the Fe-sulfides were transferred into centrifuge tubes. A final purification was carried out using an HCl and xylene rinse to remove Fe-oxides and S^0^. The samples were centrifuged at 5000 rpm for 5 min and re-suspended in DIW, 1 M HCl (2X), xylene (2X), dichloromethane (2X) and ethanol (3X), with centrifugation between each rinsing step. The purified pyrite samples were then freeze dried and stored in a vacuum desiccator.

### X-ray diffraction and X-ray fluorescence

Freeze dried smectite clay powders were analyzed using side loaded powder XRD sample holders to minimize preferred orientation of the clay minerals. Oriented mounts of the smectite clays were also prepared on zero-background silicone slides. The oriented mounts were prepared by resuspending 50 mg of clay in 200 µL of de-ionized water. The clay water suspension was pipetted onto the zero-background slides, air dried and analyzed by XRD. After analysis, the air-dried sample was saturated with ethylene glycol and heated in a sealed container at 65 °C for 4 h to expand the interlayer spaces of the smectite clays and analyzed by XRD. Synthetic pyrite samples were re-suspended in ethanol (50 mg in 200 µL) and dried on zero-background Si plates. All samples were analyzed using a Bruker D8 Advance XRD with a Cu tube and K-α radiation. A knife edge was installed to prevent scattered X-rays from entering the detector. Random powder mounts of smectites were analyzed from 5 to 65° 2Θ and oriented smectites were analyzed from 3 to 40° 2Θ. Pyrite samples were scanned from 15 to 65° 2Θ. All samples were analyzed with a step size of 0.015° at a rate of 3 s per step.

Elemental analysis of synthetic smectite and pyrite was measured using a Bruker Tiger S8 XRF instrument. Analysis of smectites by XRF was done using Li-borate glass fusions. Smectites were dried at 120 °C overnight and a 0.8 g portion was weighed out and mixed with Li-metaborate and Li-tetraborate flux (1:1 mixture of fluxes). The sample was then transferred to a platinum crucible and heated with a Claisse M4 automated fluxer that generates a glass disc for XRF analysis. The instrument was calibrated with Geological Society of Japan rock standards; JG-2, JGb-1, JB-3, JR-2, JG-1a, JA-1, JA-3, JG-3. Synthetic pyrite powders were analyzed using XRF sample cups with a 6 µm polypropylene film and analyzed with a helium purge using the Bruker XRF standard database.

### Scanning electron microscopy

Backscattered and secondary electron images of the samples were captured using a FEI Inspect F SEM instrument. The instrument is equipped with a solid-state backscattered electron detector and an Everhart–Thornley secondary electron detector. The SEM is outfitted with a Bruker XFlash 6160 60 mm^2^ silicon drift detector to perform energy dispersive X-ray spectroscopy (EDS). Synthetic pyrites were deposited onto carbon tape and directly imaged.

### Fourier transform infrared spectroscopy (FTIR)

All synthetic smectite clay mineral samples were dried at 110 °C for 18 h before FTIR spectra were collected to limit the contribution of *v*H-O–H stretching bands from water around 3400 cm^−1^ and 1630 cm^−1^. Samples were measured using an Agilent Cary 630 FTIR equipped with a 1 bounce diamond attenuated total reflectance (ATR) cell with a spectral resolution of 2 cm^−1^ from 4000 to 400 cm^−1^_._ Before each sample was analyzed the ATR cell was cleaned with ethanol, allowed to dry, and 256 background scans were run. The samples were immediately transferred onto the ATR cell and 128 scans were collected.

### Band gap measurements

Suspensions of synthetic pyrites (10 mg/mL) in de-ionized water were measured using UV–Vis-NIR spectroscopy. All spectra were collected with quartz cuvettes on an Agilent 6000i UV–Vis-NIR spectrometer scanning from 400 to 1200 nm with a 1 nm step size and an integration time of 0.1 s. Direct allowed transition band gaps were calculated with Tauc plots (α*hv*)^2^ vs. *hv*^30^.

### Smectite surface charge and particle size

Cation exchange capacity (CEC) measurements were determined using cobalt hexamine chloride (Co(III)-hexamine) absorbance at 470 nm^31^. Samples were dried (120 °C overnight) prior to measurement, then 0.2–3 g portions were mixed with 5 mL of a 30 mM Co(III)-hexamine solution. Samples were then placed in an ultrasonic water bath for 2 min, shaken for 1 h and centrifuged (5000 rpm, 30 min). Absorbance of supernatant solutions (at 470 nm) allowed measurement of molar concentrations of Co(III)-hexamine absorbed by clays to be determined. All CEC values are reported as milli-equivalents of charge per 100 g of sample (meq/100 g). Zetapotential measurements and particle size analysis of synthetic smectites were performed on 0.5% mineral suspensions in deionized water using a Malvern Panalytical Zetasizer Ultra equipped with an auto-titrator.

### Ferrous and ferric iron assay

The quantification of Fe^2+^ and Fe^3+^ from mineral suspension supernatant solutions was achieved using 1,10-phenanthroline^3^. The photochemical reduction of the Fe^3+^-phenanthroline complex can artificially produce Fe^2+^ during analysis. To prevent this, all Fe^2+^ measurements were performed under red photographic light bulbs. A 200 µl aliquot of mineral suspension was transferred into a microcentrifuge tube and spun at 13,000 rpm for 10 min. For the measurement of Fe^2+^, a 10–50 µl aliquot of supernatant was added to 50 µl of 1 wt.% 1,10-phenanthroline (dissolved in 95% ethanol). The solutions were then diluted to a final volume of 600 µl using 1 wt.% Na-citrate. The red Fe^2+^-phenanthroline complex was allowed to develop in the dark for 10 min and absorbance was measured with a UV–Vis spectrophotometer at 510 nm. The measurement of total Fe and was achieved by reducing Fe^3+^ to Fe^2+^ using hydroxylamine. A 50 µl aliquot of supernatant was added to 50 µl of 10 wt.% hydroxylamine and reacted for 5 min at room temperature. Then 50 µl of 1 wt.% 1,10-phenanthroline was added followed by dilution with 1 wt.% Na-Citrate to a 600 µl volume and measurement of absorbance at 510 nm. Standard curves were generated using ferrous sulfate heptahydrate stock solutions with concentrations ranging from 5 to 3000 µM iron.

### Hydrogen peroxide assay

Concentrations of hydrogen peroxide were quantified using methods from^3,5,32^. A buffer solution of 1 M KH_2_PO_4_ and 50 mM EDTA was prepared and adjusted to pH 4.2. A 5 mM leuco crystal violet stock solution was prepared in 0.1 M HCl. Stock solutions of horseradish peroxidase (HRP) type II were prepared in sterile deionized water at a concentration of 14.4 enzyme units/ml. Aliquots (200 µl) of mineral suspensions were transferred into microcentrifuge tubes and centrifuged at 13,000 rpm for 10 min. Then a 10 to 50 µl aliquot of the supernatant was transferred into a microcentrifuge tube containing buffer solution, achieving a final volume of 1200 µl. Next 50 µl of the leuco crystal violet reagent were added followed by 50 µl of the HRP enzyme. The samples were reacted in the dark at room temperature for 15 min. Absorbance was measured at 590 nm using a spectrophotometer and calibration curves were generated using hydrogen peroxide at 1 to 400 µM concentrations.

### Antibacterial susceptibility testing

Determination of bacterial growth inhibition vs. bactericidal activity of samples was achieved by spot plating serial dilutions of clay suspensions and cells. Colony forming units (CFU/ml) were determined using methods modified from^3,33,34^. Cultures were grown in tryptic soy broth (TSB, 30 g/L) on a shaker plate (350 rpm) at 37 °C to log-phase and diluted to ~ 10^8^ CFU/mL. The antibacterial mineral mixtures were resuspended in sterile isotonic NaCl (1.8 wt.%) and sonicated for 1 min to resuspend the minerals. Clays and bacteria were reacted at a 1:1 ratio in 12-well plates reaching a final culture volume of 2.5 mL. The 12-well plates were then incubated for 24 h on plate shaker (300 rpm) at 37 °C. Serial dilutions of samples (10^0^–10^–7^) were performed in TSB media and spot plated (10 µl) onto TSB agar plates (30 g/L) in triplicate using a multi-pipette and incubated at 37 °C for 24 h for CFU counting. Samples were considered bactericidal only if they killed 100% of cells in the undiluted sample.

Initial antibacterial susceptibility tests were performed on mixtures of F-hectorite and pyrite powders. Mixtures of synthetic smectite and 5 or 10 wt. % pyrite were ground in a mortar in pestle and transferred into 15 mL centrifuges tubes and autoclaved at 120 °C for 30 min prior to antibacterial susceptibility testing. These percentages of pyrite were similar to those observed in natural antibacterial samples^3^. The mineral mixtures were tested against *Escherichia coli* ATCC 25922 growing in TSB at concentrations of 50 and 100 mg/mL.

The antibacterial activity of mineral mixtures exchanged multiple times with Fe^2+^ in an anerobic glove box was also measured. However, the pH of the mineral suspensions decreased during the multiple Fe^2+^ exchanges and subsequent washing with DIW. Additionally, the preparation of Fe^2+^ exchanged clays in an anerobic glove box is time consuming and limits scaling the synthesis of these mineral formulations for medical or industrial applications. To mitigate these challenges a series of samples were prepared under normal atmospheric conditions using nitrogen purged solutions and limited Fe^2+^ exchange steps. Concentrations of pyrite in the mineral mixtures and Fe^2+^ used in the exchange reactions were varied to determine the minimum concentrations needed to achieve antibacterial activity, while preventing more acidic pH values. The Fe^2+^ exchange reactions in ambient atmospheric conditions were all performed in nitrogen purged solutions. Solutions of DIW were purged for 1 h with nitrogen and FeSO_4_ solutions were prepared under constant nitrogen purge. The Fe^2+^ exchange of F-hectorite pyrite mixtures was carried out in two ways. First a series of F-hectorite clays were mixed with varying concentrations of pyrite, ranging from 1.5 to 20 wt.% pyrite and exchanged once with 30 mM FeSO_4_. The second series of samples had a fixed concentration of 5 wt. % pyrite and varying concentrations of FeSO_4_ exchange solutions ranging from 5 to 60 mM FeSO_4_. All samples were Fe^2+^ exchanged once in an ultrasonic bath for 10 min in a sealed centrifuge tube. The concentration of minerals in all Fe^2+^ exchange reactions was 10 mg/mL. After 10 min of exchange the samples were centrifuged for 10 min at 3,500 rpm in a swinging bucket centrifuge. The supernatant was then decanted and an aliquot of degassed DIW was added to the centrifuge tube. The centrifuge tube was then sealed and the sample was resuspended in an ultrasonic bath for 2 min, followed by centrifugation at 3500 rpm for 10 min. The supernatant was decanted, and the samples were then resuspended in 95% ethanol in an ultrasonic bath for 2 min, followed by centrifugation at 3500 rpm for 10 min. The supernatant ethanol was decanted, and the samples were lyophilized overnight. After lyophilization, the samples were autoclaved for 30 min at 120 °C and stored in sealed centrifuge tubes for subsequent antibacterial testing, as described above.

### Antibacterial susceptibility testing of the multidrug resistant ESKAPE pathogens

Once the correct ratio of minerals (smectite and pyrite) and Fe^2+^ exchange solution that mimicked the natural antibacterial minerals were determined, the ESKAPE pathogens were tested for antibacterial susceptibility. The antibacterial mineral mixtures tested contained 95 wt.% synthetic smectite and 5 wt.% synthetic pyrite and were exchanged with 30 mM FeSO_4_, in ambient lab conditions, as described in the previous section. The minerals formulations were tested at doses of 10, 25, 50, 75 and 100 mg/mL in TSB media as described in the previous section. The multidrug resistant ESKAPE strains tested consisted of; *Enterococcus faecalis* ATCC 700802, *methicillin resistant staphylococcus aureus* ATCC 33591, *methicillin resistant staphylococcus aureus* ATCC 43300, *Klebsiella pneumoniae* ATCC 27736, *Acinetobacter *sp. ATCC 17987, *Pseudomonas aeruginosa* ATCC BAA-2114, *ESBL Escherichia coli* ATCC BAA-196 and *ESBL Escherichia coli* ATCC BAA-2326. Antibiotic susceptible strains were also tested at the same dose range (*Staphylococcus epidermidis* ATCC 14990, *Staphylococcus aureus* ATCC 25923).

### Fibroblast mineral testing

Mammalian murine fibroblasts (NIH-3T3) cells were seeded at 70% confluency in RPMI-1640 cell culture media supplemented with 10% fetal bovine serum in the presence of 1% penicillin/streptomycin (complete media). On day 2 of culture, media was exchanged for one part complete media and one part 1.8% NaCl by volume without or with 10 mg/ml and 25 mg/ml antibacterial mineral mixtures (95 wt. % smectite and 5% pyrite, exchanged with 30 mM FeSO_4_) resuspended in 1.8% NaCl. Cells were treated with respective mineral concentrations for 4 h and replaced with complete media and allowed to recover. Surviving cells were imaged in brightfield or processed for Ki67 antibody staining.

### Immunocytochemistry

Immunocytochemistry was used to assess Ki67 protein abundance to mark proliferation of surviving fibroblasts following a 4 h mineral exposure. At time of collection (4 h) or after a short term (48 h) or long term (1 week and 2 weeks) recovery in complete media, cells were washed three times with sterile saline and fixed for 20 min with 4% paraformaldehyde. Non-specific epitopes were blocked with 4% BSA in PBS for 30 min followed by incubation with Ki67 (Abcam, ab16667) at a 1:250 dilution in 4% BSA for 1 h at RT. Subsequently, an AlexaFluor488 secondary antibody (ThermoFisher Scientific, #A11008) was used at 1:500 dilution in combination with DAPI (ThermoFisher Scientific, #62247) (1:100). Cells were imaged on an Echo Revolve microscope at 10× magnification.

### Elemental analysis

Elemental analysis was performed on supernatant solutions over 24 h from mineral mixtures containing 95 wt.% synthetic F-hectorite and 5% pyrite, exchanged once with nitrogen purged 30 mM FeSO_4_ and DIW as described above. The mineral mixtures were suspended in a 1:1 mixture of isotonic NaCl (1.8 wt.%) and tryptic soy broth (30 mg/mL) at mineral concentrations of 10, 25, 50, 75 and 100 mg/mL. After reacting for 24 h at 37 °C on a shaker table in tinfoil covered flasks, the mineral suspensions were centrifuged for 1 h at 13,000 rpm. The supernatant solutions were diluted in 2% nitric acid containing 1 ng/g In, Re and Bi, and then analyzed using the Thermo Element XR high-resolution ICP-MS instrument at LLNL. To quantify raw intensities, a series of calibration standards were analyzed alongside the unknowns. These included USGS rock standards such as BCR-2 and AGV-2 and multi element calibration solutions with known concentrations. To assess the long-term accuracy and precision of this technique the USGS rock standard BHVO-2 has been analyzed multiple times from multiple digestions, with concentrations typically within 10% of reference values^35^. An aliquot of the supernatant solution was taken for fluorine analysis using a Mettler Toledo DX219-F selective ion electrode and diluted in a 1:1 ratio with TISAB III prior to analysis.

Concentrations of Li in synthetic smectites were analyzed using a selective ion monitor after digesting samples in 10 M HCl containing 4 M HF and heated at 90 °C in a Teflon vial for 4 h. After samples were digested and evaporated to near dryness, they were resuspended in 2% nitric acid and heated at 90 °C until the solution evaporated. The samples were then resuspended in de-ionized water and measured with a Mettler Toledo DX207-Li electrode.

### Mineral hydrogel antibacterial susceptibility testing

The application of mineral poultices may complicate the application of antibacterial mineral formulations used for wound care and applications that limit the release of minerals are desirable. We tested Fe^2+^ exchanged F-hectorite pyrite mixtures imbedded in 1 wt.% agarose hydrogels to determine if antibacterial activity was observed in a hydrogel matrix. Mixtures of Fe^2+^ exchanged (30 mM, ambient conditions) F-hectorite and 5 wt.% pyrite were used for the agarose hydrogel experiments. A 2 wt. % solution of agarose was prepared using DIW and microwaved until it dissolved. The agarose solution was then placed in a 65 °C water bath while the mineral mixtures were hydrated. Mineral mixtures were suspended in sterile isotonic solution (1.8 wt.% NaCl) and mixed in a 1:1 ratio with the 2 wt.% agarose solution, achieving a final agarose concentration of 1 wt.%. The suspension was immediately vortexed for 5 s and 1.5 mL was pipetted into a 12-well plate to cure. Cultures of *E. coli* ATCC 25922 and *S. epidermidis* ATCC 14990 were grown to log phase in TSB and diluted to ~ 10^8^ CFU/mL. A 1.5 mL aliquot of bacterial culture was added to the 12-well plate containing the mineral agarose composites. The final solution to mineral concentrations in each well were 100, 50, 25 and 12.5 mg/mL. Bacterial growth was monitored using Uv–Visible spectroscopy and optical density measurements at 600 nm (OD600). To measure the optical density, 200 µL of bacterial culture was sampled and pipetted into a 96-well plate and measured using a microplate reader at 0, 3 and 24 h. Spot plates were also prepared to determine if the samples were bactericidal. A 20 µL aliquot of sample was deposited onto a TSB-agar plate and incubated at 37 °C for 24 h. The pH was measured by transferring 1 mL of the culture solution into a 15 mL centrifuge tube after 24 h.

### Thermodynamic modeling

Speciation modeling of the mineral stability Pourbaix diagrams for the Fe–S–O–H system were calculated using Geochemist’s Workbench V14.0 and the MINTEQ thermodynamic database. The stability fields were calculated from infinite dilution up to a concentration of 2 mM Fe and 5 mM S, which represents the concentrations of Fe where antibacterial activity is observed. The system was modeled from pH 0 to 9 and Eh −0.75 to 1.25 V.

### Mineral fibroblast toxicity testing

The viability of 3T3 mouse fibroblasts exposed to the antibacterial mineral mixtures was measured to determine if mammalian cells could withstand the application of a mineral poultice while maintaining antibacterial characteristics. The antibacterial mineral mixtures were prepared as described previously with 95% F-hectorite clay, 5% pyrite and exchanged with 30 mM FeSO_4_ and autoclaved prior to testing. All experiments were performed in a 12-well plate using 3T3 mouse fibroblast cells grown to 85% confluency in RPMI media supplemented with 10% dialyzed fetal bovine serum. The fibroblast cells were reacted with 25 and 100 mg/mL concentrations of the antibacterial mineral mixtures in RPMI media. After 24 h of exposure, the media and minerals were decanted and the cells were rinsed with 0.8% sterile sodium chloride buffer three times to remove excess minerals. The cells were then trypsinized to detach them from the 12-well cell culture treated plate. Samples were then pelleted in a 1.5 mL centrifuge tube and stained with trypan blue to determine cell viability using a Countess automated cell counter. A control well with no cells and 100 mg/mL minerals was also included to determine if the minerals interfered with the viability assay.

## Supplementary Information

Supplementary Information.
