# Supplementary material for: Synthetic antibacterial minerals: harnessing a natural geochemical reaction to combat antibiotic resistance
Source: Sci Rep. 2022 Jan 24;12:1218. doi: 10.1038/s41598-022-05303-x (PMC8786894; doi:10.1038/s41598-022-05303-x)
Supplement: Supplementary file 1 — Supplementary Information. [file 41598_2022_5303_MOESM1_ESM.pdf]

Supplementary Information for:

Synthetic antibacterial minerals: harnessing a natural geochemical reaction to combat antibiotic resistance

Keith D. Morrison[1,a], Kelly A. Martin[2], Josh B. Wimpenny[1] and Gabriela G. Loots[2]

[1]Nuclear and Chemical Sciences Division, [2]Biosciences and Biotechnology Division, Physical and Life Sciences, Lawrence Livermore National Laboratory, Livermore, California 94550, United States

[a]**Corresponding Author Email:** morrison30@llnl.gov

This PDF file includes:

Supplementary text
Figures S1 to S10
Tables S1 to S3
SI References

Supplementary Information Text

Publication rate for research on antibacterial clays. The study of natural antibacterial clays started with the seminal paper by Williams et al., 2004 [1], revealing that natural clay minerals with no pre-treatments or alterations had intrinsic antibacterial properties. An exponential increase in publications on this research topic occurred over the next decade (Figure S1.). However, to date no study has shown that this process can be reproduced with samples that exhibit consistent reactivity, high chemical purity, and the absence of any unwanted elements.

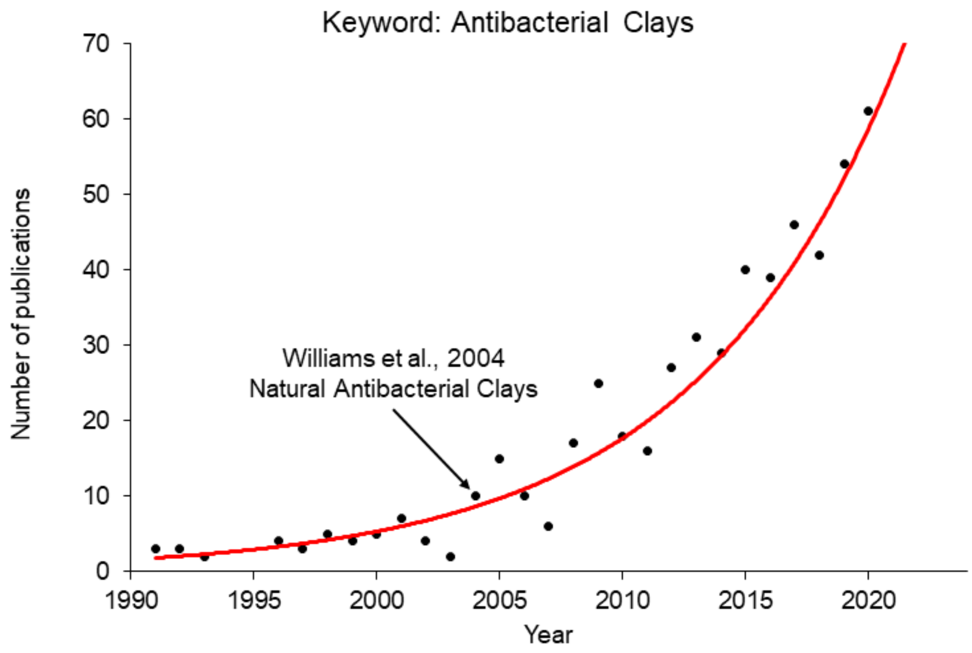

Figure S1. Number of publications per year on antibacterial clays showing an exponential increase over the last 15 years, obtained from a PubMed (https://pubmed.ncbi.nlm.nih.gov/) keyword search for antibacterial clays.

Smectite synthesis reaction conditions. Typical smectite synthesis methods require long reaction times, many times requiring 3-6 months to produce a layered 2:1 clay. Additionally, many secondary minerals (zeolites) can form during synthesis depending on the SiO_2 source, cation content and pH [2,3]. When samples were reacted at 180°C for 3 days, a portion of the fumed SiO_2 particles remained (Figure S2A). The amorphous XRD peak from the fumed SiO_2 particles was not present when samples were reacted for 5 days at 200°C (Figure 1A, main text). The basal spacing of the synthetic F-hectorite interlayer spaces (001 *hkl* peak) is 12.8 Å, which is common for smectite clay minerals (Figure 2C). Fluorine greatly increased the crystallization rate of the smectites and samples reacted without F had weak 001 (*hkl*) peaks after reacting for 5 days at 200°C, and no additional peaks associated with 2:1 expandable smectite clay mineral were observed (Figure S2B-D). Upon saturation with ethylene glycol, the interlayer space expands to 17.2 Å and *hkl* reflections, characteristic of expandable smectite clays, appear (Figure S2B). The random powder mounts show a peak at 1.52 Å that is characteristic of trioctahedral hectorite clay minerals (Figure 2A) [3].

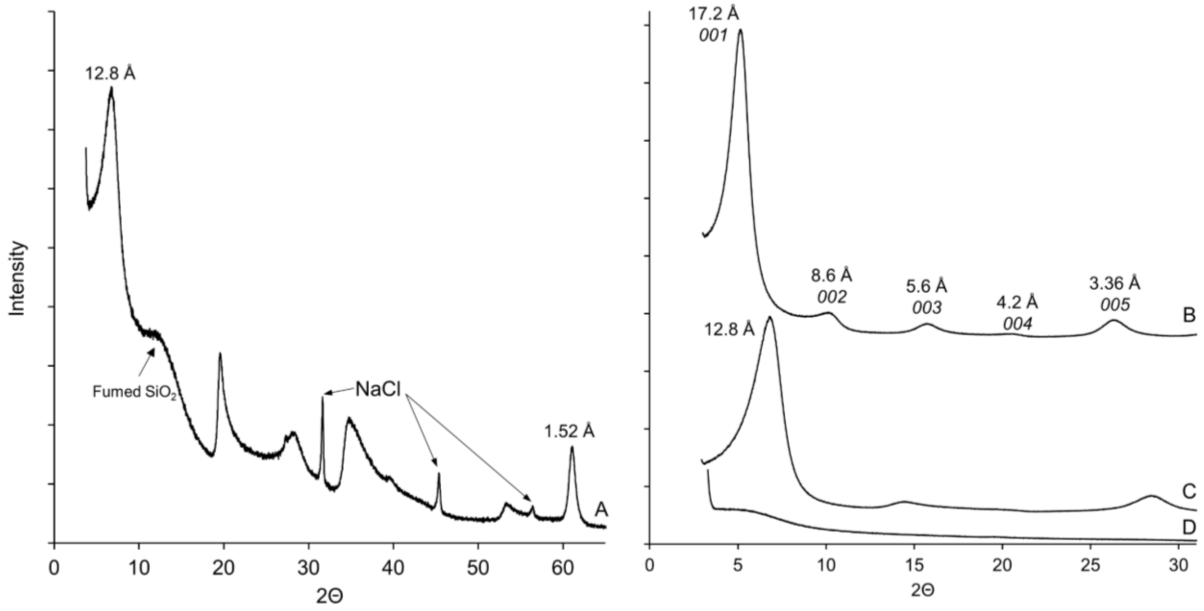

Figure S2. X-ray diffraction patterns of synthetic F-hectorites. (A) Random powder XRD pattern of synthetic smectite reacted at 180°C for 3 days, XRD peaks from NaCl are a result of incomplete dialysis of clays. Nanoparticles of SiO_2 are still present after 3 days of reaction at 180°C. Oriented clay XRD patterns of (B) ethylene glycol (EG) saturated and (C) air dried synthetic smectite after reacting for 5 days at 200°C. (B) Basal *hkl* spacing (001, 002…) of EG saturated samples are indicative of layered 2:1 smectite clay minerals. (D) Synthesis at 200°C for 5 days in the absence of fluorine did not result in the formation of smectite clay minerals.

Hectorite smectite clays have trioctahedral site occupancy with Li^+ and Mg^{2+} occupying all potential octahedral sites [4,5]. The FTIR spectra of the synthetic F-hectorites are indicative of natural hectorite clay minerals [4,5]. The FTIR spectra show octahedral stretching from Mg and Li in the octahedral sheets at 3616 cm^{-1} and 3687 cm^{-1} respectively (Figure S3, Table S1). Stretching and deformation bands from tetrahedral Si-O bonds are present at 955, 778, 700 and 412 cm^{-1} (Figure S3, Table S1) [4,5]. The amorphous fumed SiO_2 nano-particles used in the synthesis reaction have Si-O stretching and deformation bands at 1190, 1067 and 800 cm^{-1} (Figure 2). Combination Gaussian-Lorentzian curve deconvolution of FTIR bands at 1190 and 1067 cm^{-1} was used to determine the percentage of unreacted fumed SiO_2 remaining in the synthetic clays under different reaction conditions. Samples reacted at 180°C for 3 days have 24% unreacted SiO_2 nano-particles present (Figure S3). After reacting for 5 days at 200°C samples contain 4% unreacted SiO_2 nano-particles.

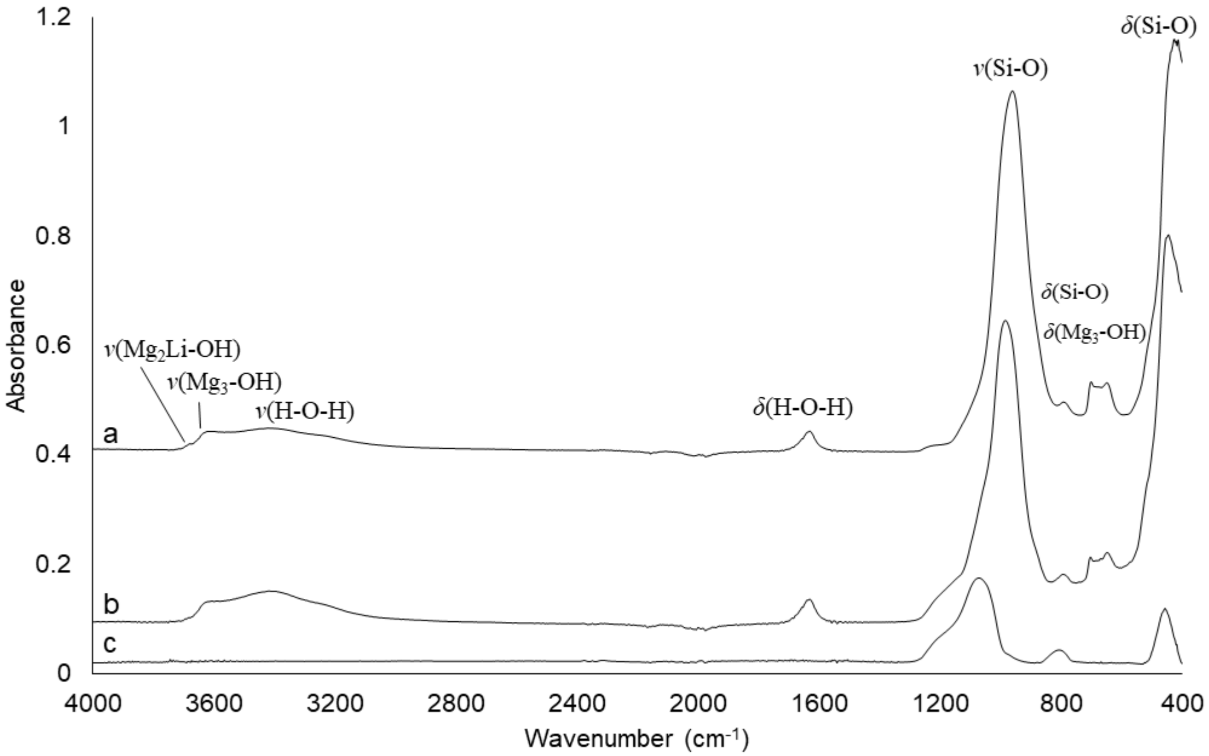

Figure S3. FTIR spectra of synthetic F-hectorite after reacting for (a) 5 days at 200°C and (b) 3 days at 180°C.(c) Spectrum from the fumed SiO_2 nano-particles used to synthesize the clays.

Table S1. FTIR band assignments for synthetic F-hectorite clays.

Wavenumber cm^{-1}	Band Assignment	Description
3687	$v(Mg_3\text{-}OH)$	Octahedral *v*OH-stretching
3616	$v(Mg_2Li\text{-}OH)$	Octahedral *v*OH-stretching
3400	*v*(H-O-H)	Adsorbed water *v*OH-stretching
1626	*δ*(H-O-H)	Adsorbed water *δ*OH-deformation
955	*v*(Si-O)	Tetrahedral *v*SiO-stretching
778	*δ*(Si-O)	Tetrahedral *δ*SiO-deformation
700	*δ*(Si-O)	Tetrahedral *δ*SiO-deformation
642	$\delta(Mg_3\text{-}OH)$	Octahedral *δ*OH-deformation
412	*δ*(Si-O)	Tetrahedral *δ*SiO-deformation

Smectite cation exchange capacity, zetapotential and particle size. The synthetic F-hectorites have a cation exchange capacity (CEC) of 98.9 ± 3.1 meq/100g (milli-equivalents of charge per 100 g of sample) (Figure S4A). The CEC values of the synthetic F-hectorites are in the range of natural 2:1 smectite clay minerals that have CEC values ranging from 60 to 120 meq/100g [6]. The fumed SiO_2 used in the synthesis reaction had almost no cation exchange capacity with a value of 0.3 meq/100g measured. Zetapotential measurements of synthetic smectites with varying pH show the clays maintain a net negative surface charge from pH 3-9. This is due to the negatively charged basal surfaces of the smectite that dominate the surface charge of the clay.

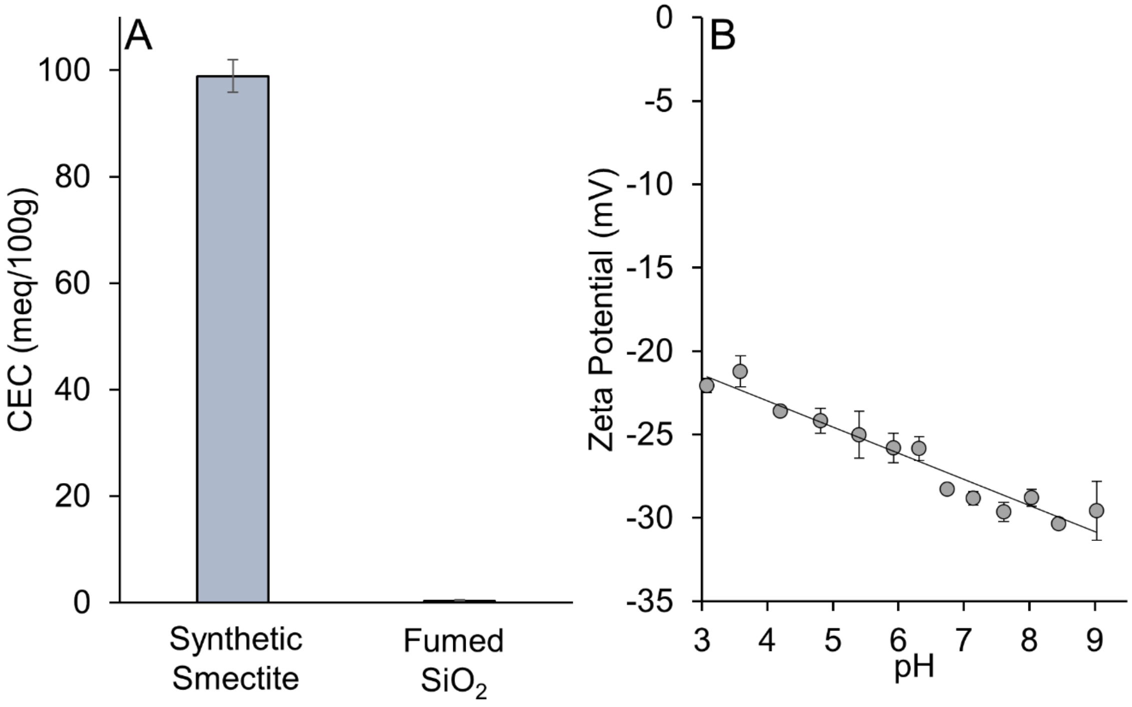

Figure S4. (A) Cation exchange capacity measured on synthetic smectite and fumed SiO_2 used to synthesize the clays. All results are reported as milli-equivalents of charge per 100 g of sample (meq/100g). (B) Zeta potential measurements of synthetic smectite measured from pH 3-9, showing a net negative charge is maintained on the smectite surfaces.

Pyrite synthesis reaction conditions and removal of elemental sulfur. Synthesis reactions using only S^0 as the sulfur source formed pyrite, however the reaction was not complete and weight % quantities of S^0 remained in the final product (Figure S5A). Additionally, the pyrite particles that formed did not have uniform size and shape (Figure S6A-B). When polysulfides were used in the pyrite synthesis reaction only minor S^0 impurities were observed in the XRD pattern (Figure S5B). Rinses with xylene effectively removed these impurities and produced pure pyrite powders (Figure S5C and S6). The SEM images of the purified pyrite samples indicate that 1-2 µm spheres form (Figure S6), similar to the size of pyrite particles observed in the natural antibacterial minerals [7].

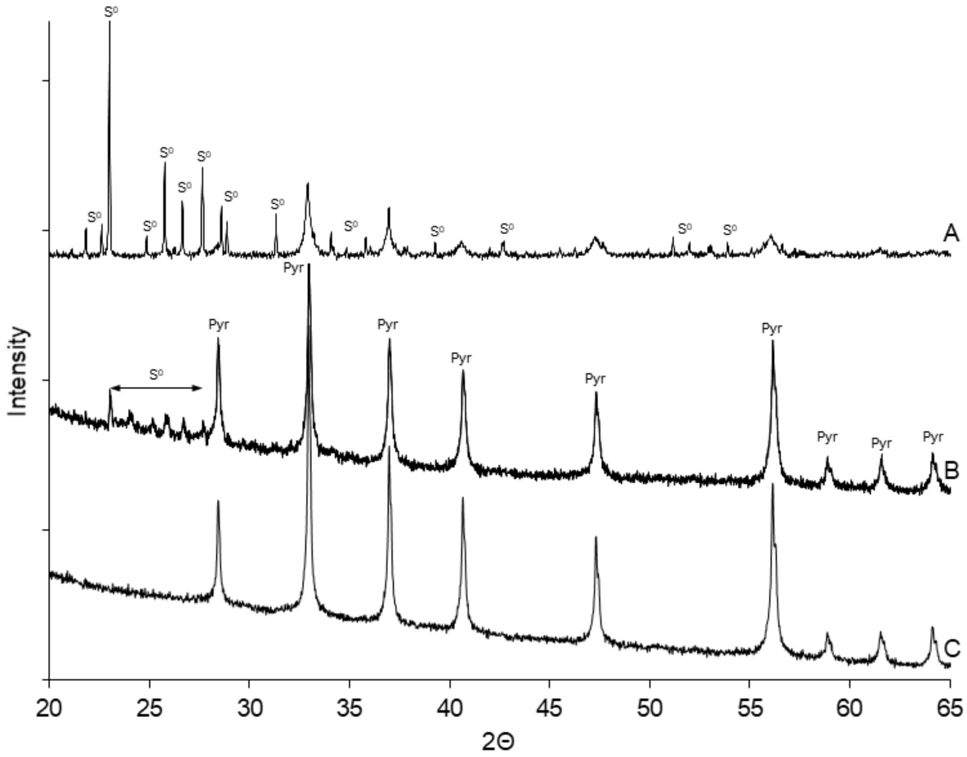

Figure S5. XRD patterns of synthetic pyrites. (A) Unreacted sulfur dominated the XRD pattern when S^0 alone was used for synthesis. (B) Synthetic pyrite produced via the polysulfide synthesis method resulted in fewer S^0 impurities that could be removed with xylene (C). Elemental sulfur peaks and pyrite peaks in XRD patterns are indicated by S^0 and Pyr, respectively.

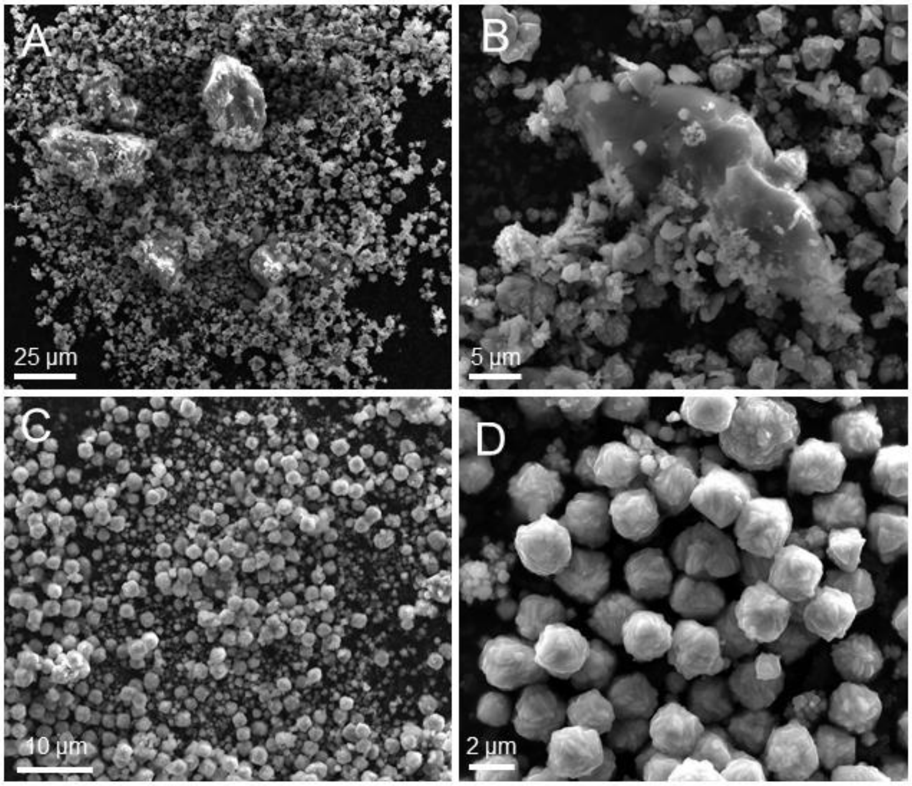

Figure S6. Scanning electron microscopy images of synthetic pyrites reacted at 200°C for 48 hours. (A-B) Pyrite synthesis using S^0 showing 10-25 µm particles of S^0 remain after reaction with pyrite spheres and flakes 0.2-2 µm in size forming. (C-D) Pyrite synthesized using polysulfides and xylene to remove trace S^0 impurities. Uniform spheres of pyrite were observed, ranging in size from 1-2 µm.

Mineral mixtures and antibacterial activity. Initial antibacterial susceptibility tests were performed on mixtures of synthetic smectite and pyrite powders. Mixtures of smectite with 5 and 10 wt. % pyrite were prepared in a mortar in pestle and transferred into 15 mL centrifuges tubes and autoclaved at 120°C for 30 minutes prior to antibacterial susceptibility testing. These percentages of pyrite were similar to those observed in natural antibacterial samples [8]. The F-hectorite-pyrite mixtures alone did not produce any antibacterial activity and levels of soluble Fe^{2+} and H_2O_2 were below detection limits (1 µM and 5 µM, respectively) (Figures S7 and S8). The pH of the smectite-pyrite mixtures was 5.5 when tested against *E. coli* (Figure S7). The pH of the solution rose to 7.1 after 24 hours as a result of bacterial growth. Antibacterial mineral mixtures observed in nature instantaneously released Fe^{2+} and generated H_2O_2 upon hydration. The cation exchange capacity of the expandable smectite clay minerals was hypothesized to be a source of Fe^{2+} that would rapidly release in solution and initiate the redox cycling reactions with pyrite [9]. Therefore, the cation exchange of synthetic smectites may result in the rapid release of Fe^{2+} which starts the redox cycling of Fe^{2+}/Fe^{3+} with pyrite surfaces while generating H_2O_2.

A series of experiments using Fe^{2+} exchanged smectites and pyrite were conducted to determine if smectite interlayer Fe^{2+} would initiate antibacterial activity. All Fe^{2+} exchange reactions were conducted in an anerobic glove box to prevent the oxidation of Fe^{2+}during sample processing. The concentration of minerals in all Fe^{2+} exchange reactions was 10 mg/mL. Samples were exchanged with 120 mM $FeSO_4$ (nitrogen purged) 3 times and centrifuged at 3500 rpm for 10 minutes between each exchange. After the Fe^{2+} exchange, the samples were rinsed with nitrogen purged DIW in the glove box 3 times, with a centrifugation step between each rinse. The samples were then resuspended in 95% ethanol and centrifuged at 3500 rpm for 10 minutes and dried under a nitrogen purge. After the samples had dried, they were autoclaved at 120°C for 30 minutes.

Mineral mixtures exchanged with Fe^{2+} containing F-hectorite and 5 wt. % pyrite immediately released 2 mM Fe^{2+} during reaction with *E. coli,* and maintained 0.5 mM concentrations of Fe^{2+} over 24 hours (Figure S7). These samples resulted in the immediate generation of 319.2 µM H_2O_2 and maintained 67.6 µM H_2O_2 over 24 hours (Figure S7). The initial and 24 hour pH values for the Fe^{2+} exchanged F-hectorite 5 wt. % pyrite mixtures were 4.2 and 3.7, respectively. Bactericidal antibacterial activity was observed after 4 hours (Figure S8). The Fe^{2+} exchanged synthetic smectites with no added pyrite immediately released 0.7 mM Fe^{2+} and maintained 75 µM Fe^{2+} over 24 hours (Figure S7). The concentration of H_2O_2 immediately released was 79.2 µM and 7.6 µM H_2O_2 over 24 hours, with initial and 24 hour pH values of 4.9 and 4.4, respectively (Figure S7). The Fe-hectorite Fe^{2+} exchanged samples were not bactericidal, however they did inhibit bacterial growth over 24 hours (Figure S8). A mixture of Fe^{2+} exchanged F-hectorite containing 20 wt. % pyrite was also tested for antibacterial activity, Fe^{2+} and H_2O_2 generation to determine if increased pyrite concentrations could extend the duration of the ROS generating reactions. These samples resulted in the instant release of 1.5 mM Fe^{2+} (Figure S7). After 4 hours a drop in Fe^{2+} concentrations was observed, similar to the Fe^{2+} exchanged F-hectorite with or without 5 wt. % pyrite. However, after 24 hours the levels of Fe^{2+} increased to 3.6 mM in the samples containing 20 wt. % pyrite. The H_2O_2 concentrations followed a similar trend with initial concentrations of 319.2 µM H_2O_2 measured, increasing to 464.8 µM H_2O_2 after 24 hours (Figure S7). The pH values for the 20 wt. % pyrite samples were lower, with values of 3.5 and 2.8 measured initially and after 24 hours, respectively. These samples were bactericidal against *E. coli* after 4 hours, however the pH decreased to 2.8 in these samples which is bactericidal to *E. coli* and *S. epidermidis* without the presence of Fe^{2+} and H_2O_2 (Figures S7 and S8). The differences in Fe^{2+} release, H_2O_2 generation and pH are discussed in the next section.

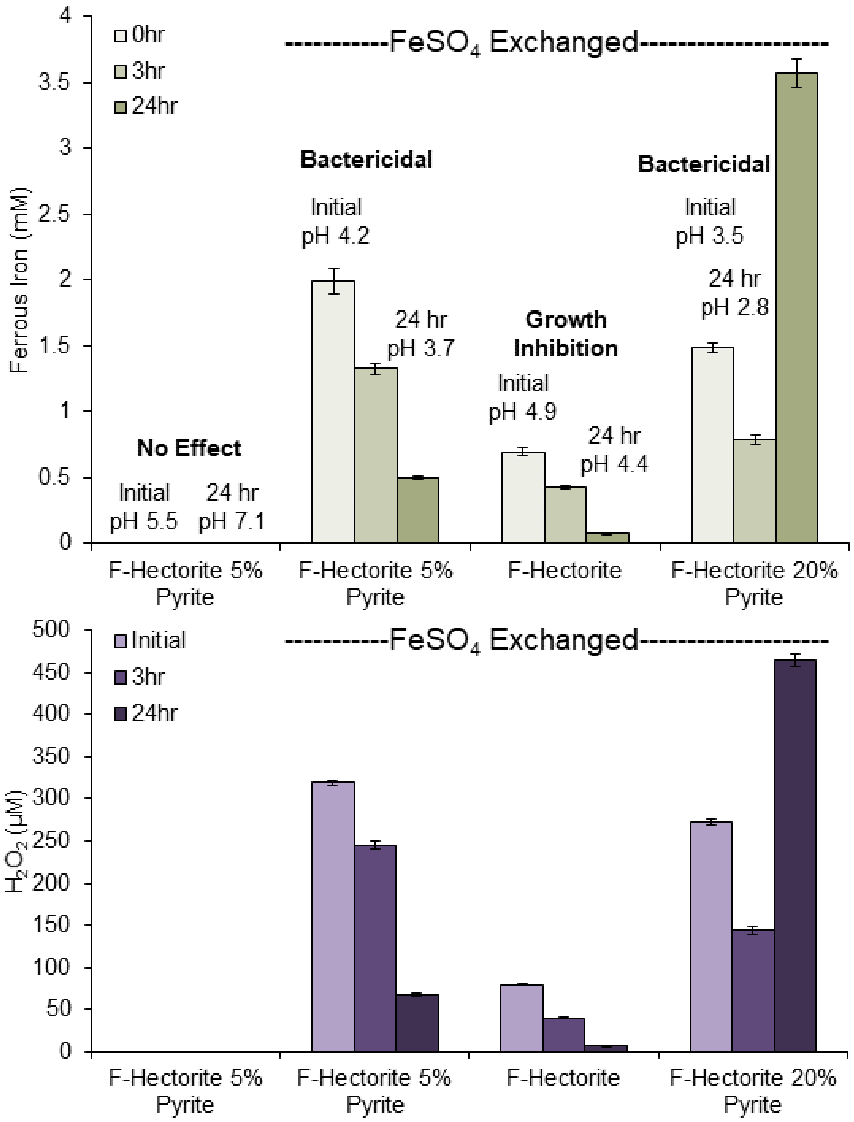

Figure S7. The influence of pyrite and smectite interlayer Fe^{2+} on pH, Fe^{2+}, H_2O_2 release and antibacterial activity. All samples were tested for antibacterial activity against *E. coli* (ATCC 25922) growing in TSB broth using 50 mg/mL mineral suspensions.

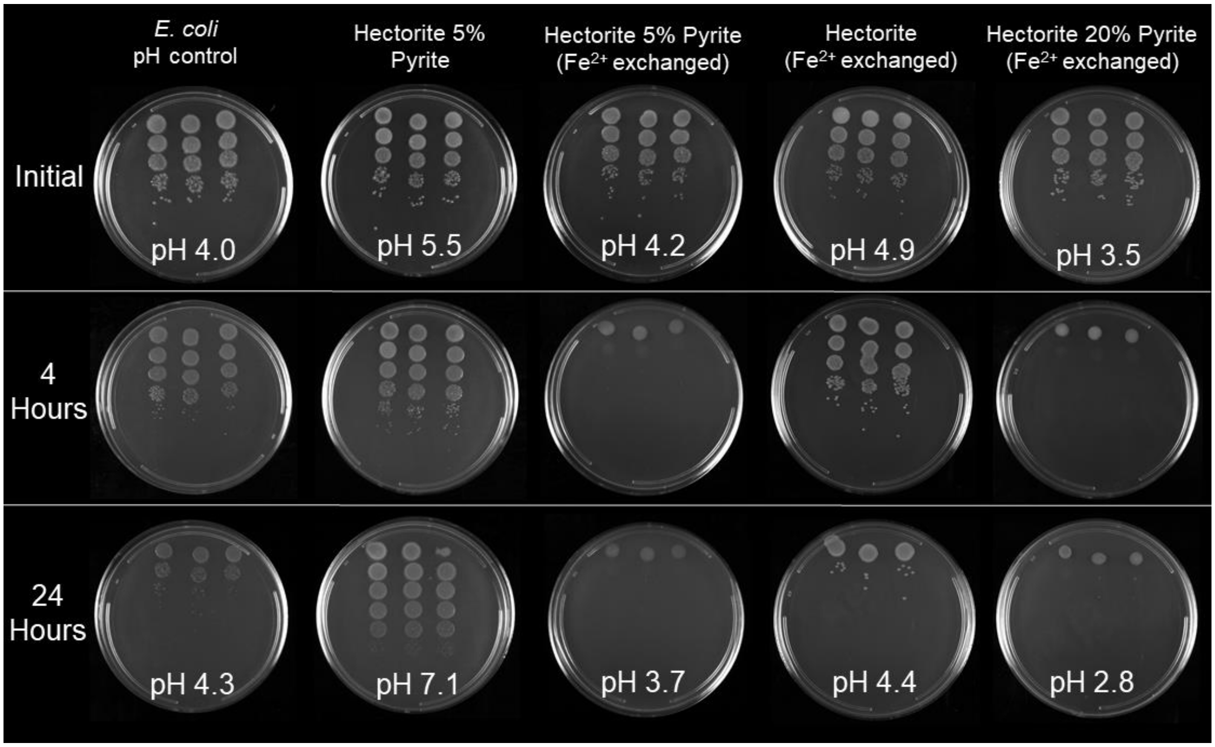

Figure S8. Antibacterial susceptibility testing (*E. coli* ATCC 25922) dilution spot plates. Mixtures of F-hectorite and pyrite alone were not antibacterial. Exchanging samples with Fe^{2+} resulted in antibacterial activity. The Fe^{2+} exchanged samples containing pyrite were bactericidal, while the Fe^{2+} exchanged F-hectorites with no pyrite inhibited bacterial growth after 24 hours.

The samples containing 20 wt. % pyrite initially released 25 % less Fe^{2+} when compared to the samples with 5 wt. % pyrite (Figure S7). The immediate release of Fe^{2+} is predominately from the smectite interlayer spaces as the samples containing no pyrite release Fe^{2+} at 0.7 mM concentrations upon hydration (Figure S7). However, the overall extent of Fe^{2+} release in the pyrite exchanged samples is greater when compared to the pure Fe^{2+} exchanged smectites, despite the pyrite exchanged samples having lower wt. % clay content. The difference in Fe^{2+} release is a result of redox reactions driving pyrite oxidation Fe^{2+} release and H_2O_2 generation [8,10]. This initial set of experiments reveal that redox cycling and cation exchange reactions taking place between the smectite clay minerals. The pyrite concentration can be used to alter the intensity and duration of Fe^{2+} release and H_2O_2 generation over 24 hours. However, acidity is a consequence of pyrite oxidation and hydrolysis of Fe^{3+} cations during the oxidation of antibacterial clays [9] and pH values decreased below 3, which may limit medicinal applications of these antibacterial clays. Limited Fe^{2+} exchange and DIW rinsing of the samples prevented the drop in pH and allowed the synthetic antibacterial mineral assemblages to buffer pH in the range of 5.5-3.7 over 24 hours, depending on the mineral concentration (Figure 2C, main text).

The initial antibacterial testing experiments above reveal that pyrite concentration and Fe^{2+} cation exchange of smectites can influence the antibacterial activity of synthetic minerals mixtures. We varied these two factors to test the degree of control they have on antibacterial activity. All samples were tested for antibacterial activity, Fe^{2+}, H_2O_2 and pH as previously described (using *E. coli* and TSB broth), with dilution spot plates measured after 4 and 24 hours (Figures 9-11). Mineral mixtures with varying pyrite (1.5 to 20 wt. %) and a fixed Fe^{2+} exchange solution concentration (20 mM) reveal that increases in pyrite loading directly correlate with increased concentrations of Fe^{2+} and H_2O_2 measured in solution (Figure

9a). The concentrations of Fe^{2+} released after 1 hour ranged from 0.76 mM in samples containing 1.5 wt. % pyrite, to 7.9 mM in samples containing 20 wt. % pyrite (Figure 1F). The concentrations of H_2O_2 ranged from 77 to 300 µM in samples with 1.5 and 20 wt. % pyrite, respectively (Figure 1F). The pH values in these samples were higher with values ranging from 5.45 to 4.7 with pyrite ranging from 1.5 to 20 wt. %, respectively (Figure 1F). The mineral mixtures became bactericidal when pyrite concentrations were ≥ 10 wt. % and Fe^{2+}, H_2O_2 and pH concentrations were ≥ 4 mM, 209.5 µM and 4.96, respectively. These results indicate that acidity from metal hydrolysis and pyrite oxidation occurred when samples were processed with multiple Fe^{2+} exchanges in a glove box. The presence of Fe^{3+} in solution can initiate pyrite oxidation even in the absence of oxygen and generate acidity through hydrolysis reactions and pyrite oxidation. Limiting the Fe^{2+} exchange and rinsing steps prevent excess acid from accumulating.

A series of synthetic smectite-pyrite mixtures were also prepared with a fixed pyrite concentration (5 wt. %) and varying concentrations of Fe^{2+} exchange solution (5 to 60 mM) (Figure 1G). These samples released 1.3 to 3.5 mM Fe^{2+} and 161.1 to 274.1 µM H_2O_2 after 4 hours (Figure 1G). The increasing concentrations of Fe^{2+} and H_2O_2 released correlate with increases in the concentration of Fe^{2+} exchange solution as expected. The pH in this series of samples was also higher with values ranging from 5.22 to 4.75 in samples exchanged with 5 to 60 mM Fe^{2+} (Figure 1G). Samples exchanged with ≥ 30 mM Fe^{2+} were bactericidal when Fe^{2+}, H_2O_2 and pH concentrations were ≥ 2.4 mM, 221.1 µM and 4.91, respectively (Figure 1G).

The multiple DIW rinsing steps used in the initial exchange reaction, carried out in an anerobic glove box to limit oxidation, resulted in lower overall Fe^{2+} concentrations and pH values < 4.2. Limited Fe^{2+} exchange reactions and rinsing steps performed in ambient atmospheric conditions still produced minerals with antibacterial properties and resulted in more basic pH values (< 5.5). Furthermore, these results show that the use of an anaerobic glove box and multiple Fe^{2+} exchange reactions are not required to produce antibacterial mineral formulations that generate extended release Fe^{2+} and ROS.

Table S2. ICP-MS elemental analysis of mineral leachates (mg/mL) reacted in TSB media (15 g/L) for 24 hours. All elemental concentrations are reported in micro-molarity (µM), with values below detection limit listed as BDL. The minimum bactericidal concentration of elements are listed along with the corresponding species tested for antibacterial activity.

Element	10 mg/mL	25 mg/mL	50 mg/mL	75 mg/mL	100 mg/mL	MBC	Species
Li	888.302	2004.107	3533.273	4816.421	6389.162	> 500,000[a]	Li^{+}
Be	0.023	0.021	0.062	0.014	0.014	131,000[d]	Be^{2+}
F	0.259	0.418	0.599	0.590	0.613	> 250,000[a]	F^{-}
Mg	1761.936	2912.208	5708.298	7657.035	9474.196	> 500,000[a]	Mg^{2+}
Al	1.139	0.190	3.253	6.431	8.521	19,000[b]	Al^{3+}
Si	788.930	934.674	1360.129	1842.067	2273.316	>100,000[a]	SiO_2
P	9268.550	6882.676	2238.456	838.326	574.833		
S	6660.158	8898.342	13067.150	18573.569	24570.131		
K	23643.589	23002.297	16810.670	16978.598	17308.626	>1,000,000[d]	K^{+}
Ca	38.160	38.133	49.818	53.360	57.147	1,500,000[e]	Ca^{2+}
Sc	BDL	BDL	0.093	0.114	0.149	6,250[c]	Sc^{3+}
Ti	0.008	0.014	0.383	BDL	BDL		
V	0.019	0.016	0.030	0.013	0.016		
Cr	0.085	0.073	0.085	0.088	0.092	300*	CrO_4^{2-}
Mn	2.074	4.178	18.619	29.074	38.874	199,000[b]	Mn^{2+}
Fe	180.277	435.462	1327.714	3901.563	6639.840	>9,000[a]/5,000[e]	Fe^{2+}/Fe^{3+}
Co	0.126	0.104	0.089	0.082	0.097	1,000[c]	Co^{2+}
Ni	0.199	0.233	0.275	0.290	0.320	18,000[b]	Ni^{2+}
Cu	0.082	0.064	0.060	0.028	0.035	15,000[b]	Cu^{2+}
Zn	2.829	1.245	3.023	3.411	3.840	31,000[b]	Zn^{2+}
Ga	0.002	0.002	0.014	0.001	0.001	25,000[d]	Ga^{3+}
Rb	1.938	1.684	1.118	0.865	0.737	66,000[d]	Rb^{+}
Sr	0.090	0.055	0.110	0.165	0.202	>200,000[d]	Sr^{2+}
Y	0.001	BDL	BDL	BDL	BDL	6,250[d]	Y^{3+}
Zr	0.008	0.014	0.003	0.001	0.001	6,250[d]	Zr^{4+}
Nb	0.002	0.001	0.001	0.001	0.001		
Mo	0.103	0.117	0.110	0.177	0.241	102,000[b]	MoO_4^{2-}
Ag	0.001	BDL	0.001	0.001	0.001	90[b]	Ag^{+}
Cd	0.003	0.002	0.004	0.004	0.006	2,300[b]	Cd^{2+}
Sn	0.003	0.003	0.018	0.003	0.004	17,000[b]	Sn^{2+}
Sb	0.014	0.011	0.008	0.005	0.003		
Cs	0.012	0.011	0.011	0.010	0.009	>48,000[d]	Cs^{+}
Ba	0.039	0.006	0.004	0.007	0.022	>32,000[d]	Ba^{2+}
La	BDL	BDL	BDL	BDL	BDL	6,250[d]	La^{3+}
Ce	BDL	BDL	BDL	BDL	BDL		

Pr	BDL	BDL	BDL	BDL	BDL		
Sm	BDL	BDL	0.001	BDL	BDL		
Eu	BDL	BDL	BDL	BDL	BDL		
Gd	BDL	BDL	BDL	BDL	BDL		
Tb	BDL	BDL	BDL	BDL	BDL		
Dy	BDL	BDL	BDL	BDL	BDL	6,250[d]	Dy^{3+}
Ho	BDL	BDL	BDL	BDL	BDL		
Er	BDL	BDL	BDL	BDL	BDL		
Tm	BDL	BDL	BDL	BDL	BDL		
Yb	BDL	BDL	0.001	BDL	BDL		
Lu	BDL	BDL	BDL	BDL	BDL		
Hf	0.001	0.001	BDL	BDL	BDL		
Ta	BDL	BDL	BDL	BDL	BDL		
W	0.003	0.002	0.002	0.001	0.001	66,000[b]	WO_4^{2-}
Tl	0.002	0.002	0.002	0.001	0.001	>134,000[d]	Tl^{+}
Pb	0.006	0.004	0.003	0.002	0.002	4,900[c]	Pb^{2+}
Th	BDL	BDL	BDL	BDL	BDL		
U	BDL	BDL	BDL	BDL	BDL		

Minimum bactericidal concentrations (MBC) and associated elemental and bacterial species are also reported from; [a]this study (*E. coli BAA196*), [b]Harrison et al., 2004 (*E. coli*) [11], [c]Harrison et al., 2005 (*E. coli*) [d]Workentine et al., 2008 (*P. fluorescens*) [12], [e]Morrison et al., 2016 (*E. coli*)[9].

Table S3. Synthetic mineral minimum bactericidal concentrations (MBC) measured over 24 hours. Minerals were tested at concentrations of 10, 25, 50, 75, and 100 mg/mL.

	Time (Hours)			
	1	4	8	24
Gram-negative	MBC Mineral Concentration (mg/mL)			
Escherichia coli BAA-2326	100	75	50	25
Escherichia coli BAA-196	>100	75	50	25
Enterobacter cloacae BAA-2468	100	75	50	50
Acinetobacter sp. 17987	50	25	25	25
Klebsiella pneumoniae 27736	75	75	75	50
Pseudomonas aeruginosa BAA-2114	75	50	50	25
Gram-positive	MBC Mineral Concentration (mg/mL)			
Staphylococcus epidermidis 14990	>100	25	25	10
Staphylococcus aureus 25923	50	25	25	25
MRSA 33591	25	25	10	10
MRSA 43300	50	25	25	25
Enterococcus faecalis 700802	100	50	50	25

Agarose hydrogel antibacterial mineral composites. The results from the agarose mineral composites tests against *E. coli* reveal show antibacterial activity when mineral concentrations reach 100 mg/mL (Figure 9a). At this concentration, the samples were bactericidal after 24 hours. Concentrations below 100 mg/mL did not show any antibacterial activity or growth inhibition for *E. coli* cultures. The pH of the antibacterial 100 mg/mL sample dropped to 4.7 after 24 hours (Figure 9b). The other mineral concentrations (50 – 12.5 mg/mL) had pH values similar to the *E. coli* control (Figure 9b). The *S. epidermidis* cells were more sensitive to the mineral agarose composites and bactericidal activity was observed at 50 mg/mL concentrations after 4 hours (Figure 9c). Concentrations of 25 mg/mL resulted in growth inhibition over 24 hours, with pH values measured at 7.1 (Figure 9d). The bactericidal concentrations (≥ 50 mg/mL) had pH values ≤ 4.7 after 24 hours. These results show that antibacterial activity is maintained when minerals are imbedded in agarose hydrogels, and the release of minerals is prevented allowing cell growth to be monitored with UV-Vis spectroscopy.

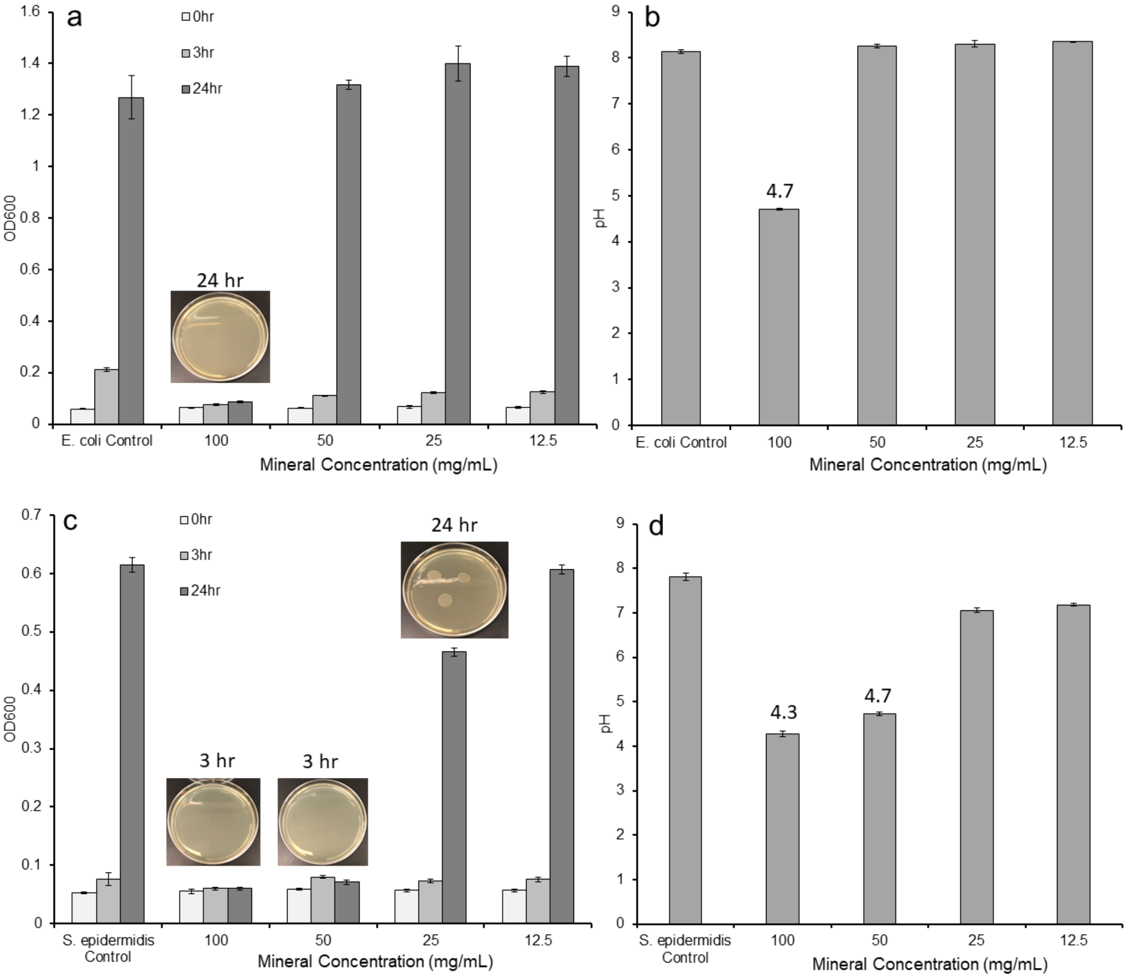

Figure S9. Antibacterial susceptibility testing of mineral agarose composites. (a) Optical density (OD) measurements (600 nm) monitoring *E. coli* growth during exposure to mineral hydrogels with varied mineral loading (100 - 12.5 mg/mL) and (b) pH values after 24 hours. (c) OD600 and pH values for mineral mixtures reacted with *S. epidermidis* and (d) pH values after 24 hours.

Toxicity of antibacterial mineral mixtures to dermal fibroblasts. The results from the fibroblast viability assay indicate fibroblast cells remain viable after a 24 hour exposure to the antibacterial minerals with approximately 93% of the cells remaining viable. The fibroblast controls with no added minerals maintained 98% viability over 24 hours (Figure S10). However, when the minerals with no fibroblast cells were measured on the automated cell counter, a viability of 31% was measured (Figure S10). This indicates that the minerals, to a degree, interfere with the trypan blue and subsequent automated cell counter viability measurement. Subtracting the influence of minerals from the fibroblast cells exposed to the antibacterial minerals indicates that approximately 62% of the fibroblast cells are viable after exposure. These results reveal that fibroblast cells are capable of surviving the toxicity produced from the antibacterial minerals. The application of antibacterial minerals to treat topical bacterial infections will require careful monitoring of the wound environment to determine when the bacterial infection is eradicated so that the minerals can be removed and wound healing can proceed.

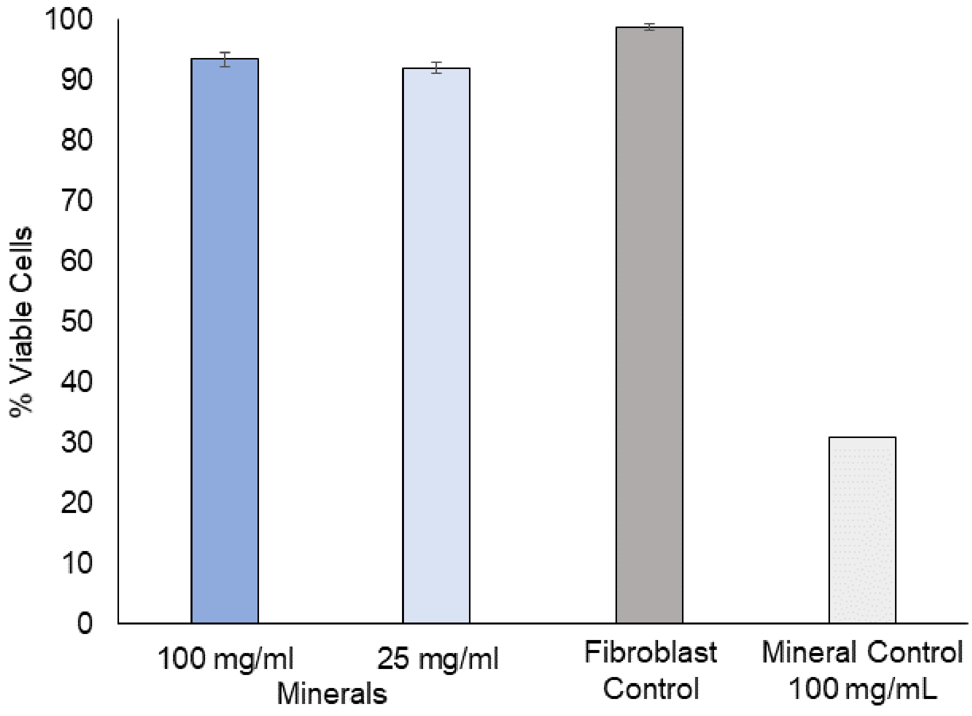

Figure S10. Fibroblast toxicity assay during 24 hour exposure to antibacterial mineral mixtures using an automated cell counter and trypan blue viability assay. Viability of 3T3 mouse fibroblasts exposed to syn-hectorite 5% pyrite Fe^{2+} exchanged minerals at 100 and 25 mg/mL concentrations after 24 hours, compared to fibroblast control with no minerals added and mineral control with no fibroblast cells.

SI References

1 Williams, L. B., Holland, M., Eberl, D. D., Brunet, T. & De Courrsou, L. B. Killer clays! Natural antibacterial clay minerals. *Mineralogical Society Bulletin*, 3-8 (2004).

2 Andrieux, P. & Petit, S. Hydrothermal synthesis of dioctahedral smectites: The Al-Fe3+ chemical series Part I: Influence of experimental conditions. *Applied Clay Science* **48**, 5-17, doi:10.1016/j.clay.2009.11.019 (2010).

3 Kloprogge, J., Komarneni, S. & Amonette, J. Synthesis of smectite clay minerals: A critical review. *Clays and Clay Minerals* **47**, 529-554, doi:10.1346/CCMN.1999.0470501 (1999).

4 Kloprogge, J. T. in *Developments in Clay Science* Vol. 8 (eds W. P. Gates, J. T. Kloprogge, J. Madejová, & F. Bergaya) 222-287 (Elsevier, 2017).

5 Kloprogge, J. T. (Clay Minerals Society, 2005).

6 Mermut, A. & Lagaly, G. Baseline studies of The Clay Minerals Society Source Clays: Layer-charge determination and characteristics of those minerals containing 2 : 1 layers. *Clays and Clay Minerals* **49**, 393-397, doi:10.1346/CCMN.2001.0490506 (2001).

7 Williams, L. *et al.* What Makes a Natural Clay Antibacterial? *Environmental Science & Technology* **45**, 3768-3773, doi:10.1021/es1040688 (2011).

8 Morrison, K., Williams, S. & Williams, L. The Anatomy of an Antibacterial Clay Deposit: A New Economic Geology. *Economic Geology* **112**, 1551-1570, doi:10.5382/econgeo.2017.4521 (2017).

9 Morrison, K., Misra, R. & Williams, L. Unearthing the Antibacterial Mechanism of Medicinal Clay: A Geochemical Approach to Combating Antibiotic Resistance. *Scientific Reports* **6**, doi:10.1038/srep19043 (2016).

10 Schoonen, M., Harrington, A., Laffers, R. & Strongin, D. Role of hydrogen peroxide and hydroxyl radical in pyrite oxidation by molecular oxygen. *Geochimica Et Cosmochimica Acta* **74**, 4971-4987, doi:10.1016/j.gca.2010.05.028 (2010).

11 Harrison, J. J., Turner, R. J. & Ceri, H. High-throughput metal susceptibility testing of microbial biofilms. *BMC Microbiol* **5**, 53, doi:10.1186/1471-2180-5-53 (2005).

12 Workentine, M. L., Harrison, J. J., Stenroos, P. U., Ceri, H. & Turner, R. J. Pseudomonas fluorescens' view of the periodic table. *Environ Microbiol* **10**, 238-250, doi:10.1111/j.1462-2920.2007.01448.x (2008).
